# Dual regulatory role of hsa-miR-122b-5p in chikungunya virus infection via interaction with CHIKV 3′-UTR and HDAC4 modulation

**DOI:** 10.1128/jvi.02118-24

**Published:** 2025-09-16

**Authors:** Priyanshu Srivastava, Nimisha Mishra, Sunil Kumar Dubey, Jatin Shrinet, Sakshi Chaudhary, Ankit Kumar, Ramesh Kumar, Surbhi Malhotra, Miguel Mano, Luca Braga, Binuja Varma, Mauro Giacca, Sujatha Sunil

**Affiliations:** 1 Vector-Borne Diseases Group, International Centre for Genetic Engineering and Biotechnology (ICGEB) 18470 , New Delhi, India; 2 Tata Consultancy Services 585536, New Delhi, India; 3 Molecular Medicine Laboratory, International Centre for Genetic Engineering and Biotechnology (ICGEB) 18470 , Trieste, Italy; University of North Carolina at Chapel Hill, Chapel Hill, North Carolina, USA

**Keywords:** chikungunya virus (CHIKV), miR-122b-5p, HDAC4, ISGs, IRF3, p-IRF3, antiviral response, macrophages

## Abstract

Establishment of a viral infection in the host involves complex interactions between the virus and the host. The present study was undertaken to evaluate one such interaction between a host microRNA (miRNA) with both the virus and its cellular target. Through a comprehensive high-throughput screen of a human miRNA mimics library, we identified that hsa-miR-122b-5p bound to chikungunya virus (CHIKV) 3′-untranslated region (3′-UTR), which we abrogated upon mutating the binding sites of the miRNA and determined that this miRNA regulated CHIKV infection. In-depth scrutiny of this miRNA’s expression in CHIKV-susceptible host cells divulged that it was mainly expressed in macrophages, and temporal expression profiling analysis in these cells revealed that its expression negatively correlated to the virus infection. We further identified cellular targets of miR-122b-5p through global RNA seq analysis using antagomiRs, among which histone deacetylase 4 (HDAC4) displayed attributes that promoted CHIKV infection in macrophages. To dissect this phenomenon further, we performed loss-of-function assays using both miR-122b-5p and HDAC4 and observed regulation of pro-inflammatory cytokines, including the type I interferon system. Furthermore, miRNA mimics and antagomiR assays indicated that regulation of HDAC4 by miR-122b-5p may be associated with changes in the nuclear translocation of phosphorylated IRF3 during CHIKV infection. While this suggests a possible link, additional studies are needed to confirm this interaction and its underlying mechanism. Taken altogether, our study provides insights into the dual regulatory function of miR-122b-5p during CHIKV infection, highlighting its capacity to decrease viral titer by binding directly to the 3′UTR of the viral genome and its potential to promote nuclear translocation of phosphorylated IRF3, which may be indirectly influenced by modulation of its cellular target, HDAC4, thereby regulating host immune responses during CHIKV infection in macrophages. These findings may have significant implications for understanding the pathogenesis of CHIKV and for developing novel therapeutic strategies.

**IMPORTANCE:**

Human microRNAs (miRNAs) are central regulators of viral infection, acting through direct targeting of viral genomes or by modulating host cellular pathways. In this study, we demonstrate that miR-122b-5p plays a dual antiviral role during chikungunya virus (CHIKV) infection in macrophages—critical immune cells that serve as viral reservoirs. We show that miR-122b-5p directly binds to the 3′ untranslated region of the CHIKV genome, thereby suppressing viral replication. Additionally, we uncover a previously uncharacterized mechanism in which miR-122b-5p modulates the host innate immune response by repressing HDAC4, a negative regulator of the type I interferon pathway. This repression may influence nuclear translocation of phosphorylated IRF3, potentially enhancing the antiviral transcriptional response. Our findings not only deepen our understanding of macrophage-intrinsic antiviral defenses against CHIKV but also highlight miR-122b-5p as a potential therapeutic target for enhancing host immunity and limiting viral propagation.

## INTRODUCTION

Macrophages, as part of the immune system, defend the host against viral infections through multiple mechanisms. Primarily, upon viral infection, macrophages boost the expression of inflammatory cytokines, triggering a strong antiviral response. Macrophages also respond to viral stimuli by activating transcription factors such as interferon regulatory factors (IRFs) and nuclear factor-kappa B (NF-κB), which induce interferon (IFN) production. This, in turn, creates a feedback loop that promotes the expression of interferon-stimulated genes (ISGs), enhancing the antiviral defense (1–3). Concomitantly, macrophages are also reported to serve as reservoirs for viruses, allowing them to persist in the cells and thereby directly contributing to progression of infection (4, 5). The underlying mechanism of this phenomenon is poorly understood, with several cellular factors probably contributing to the survival of the virus in the macrophages.

MicroRNAs (miRNAs) are vital non-coding RNA molecules involved in the post-transcriptional regulation of gene expression (6). These molecules can regulate both the host and virus in two ways: firstly, they can directly bind to the viral genome and inhibit the virus’s ability to replicate or modify its pathogenic behavior, and secondly, they can target cellular mRNAs, thereby manipulating the host cell’s machinery to counteract the virus (7–9). These molecules are known to exhibit tight regulation of their targets, which is essential for maintaining a balance between effective viral defense and avoiding excessive immune response that could harm the host. In the milieu of a viral infection, the host cell’s miRNA expression undergoes profound alterations, with these fluctuations being temporally orchestrated and meticulously regulated to either aid in host defense mechanisms or facilitate viral replication (10). For instance, studies on human macrophages have reported temporal changes in miRNA expression during the course of an infection (11, 12)—ranging from as early as the initial viral contact with the cell (13, 14) to exerting influence on the adaptive immune response during the later phases of infection. Additionally, these studies have demonstrated how these molecules can regulate cell survival and apoptosis, while also directly interacting with viral components to influence viral replication dynamics (15–17). Nonetheless, the exact temporal dynamics of miRNA modulation during viral infection can be influenced by several factors, including the specific viral pathogen, the host cell type, and individual host factors (18, 19).

CHIKV, a single-stranded RNA virus from the *Togaviridae* family, contains two coding regions split by a non-coding area and surrounded by untranslated regions (3′-UTR and 5′-UTR). The 5′-UTR initiates translation, while the 3′-UTR varies in length and aids virus adaptability and evolution (20). Studies have observed changes in microRNA (miRNA) regulation during CHIKV infection in both mammalian hosts and vectors (21–24). Furthermore, the variable 3′-UTR of CHIKV has been reported to interact with host miRNAs, which are crucial in controlling the viral replication cycle and pathogenesis (25–27).

The present study was undertaken to systematically assess the influence of human microRNAs (miRNAs) on the infection dynamics of CHIKV. Utilizing a luminescence-dependent high-throughput screening method with a miRNA-mimic library, we identified endogenous host miRNAs capable of directly interacting with the CHIKV 3'-untranslated region (3′-UTR). The expression dynamics of one such miRNA, miR-122b-5p, was observed to modulate in accordance with CHIKV infection within macrophages. Furthermore, we identified macrophage host targets of miR-122b-5p, one of them being histone deacetylase 4 (HDAC4). Further investigation of the cellular target uncovered a role for this histone deacetylase in modulating pro-inflammatory cytokine production. Silencing and overexpressing miR-122b-5p and/or HDAC4 in THP-1 cells during CHIKV infection revealed a possible role of miR-122b-5p in influencing nuclear translocation of IRF3 during infection, possibly through its regulation of HDAC4, thereby potentially contributing to an IFN α/β-induced antiviral response during the infection. Taken altogether, our data emphasize miR-122b-5p as a potent regulatory entity in macrophages during CHIKV infection and operate through a dual mechanism, directly targeting viral RNA and indirectly orchestrating the cellular antiviral response.

## RESULTS

### Human miR-122b-5p targets CHIKV 3′-UTR and negatively regulates CHIKV infection

**Fig 1:**
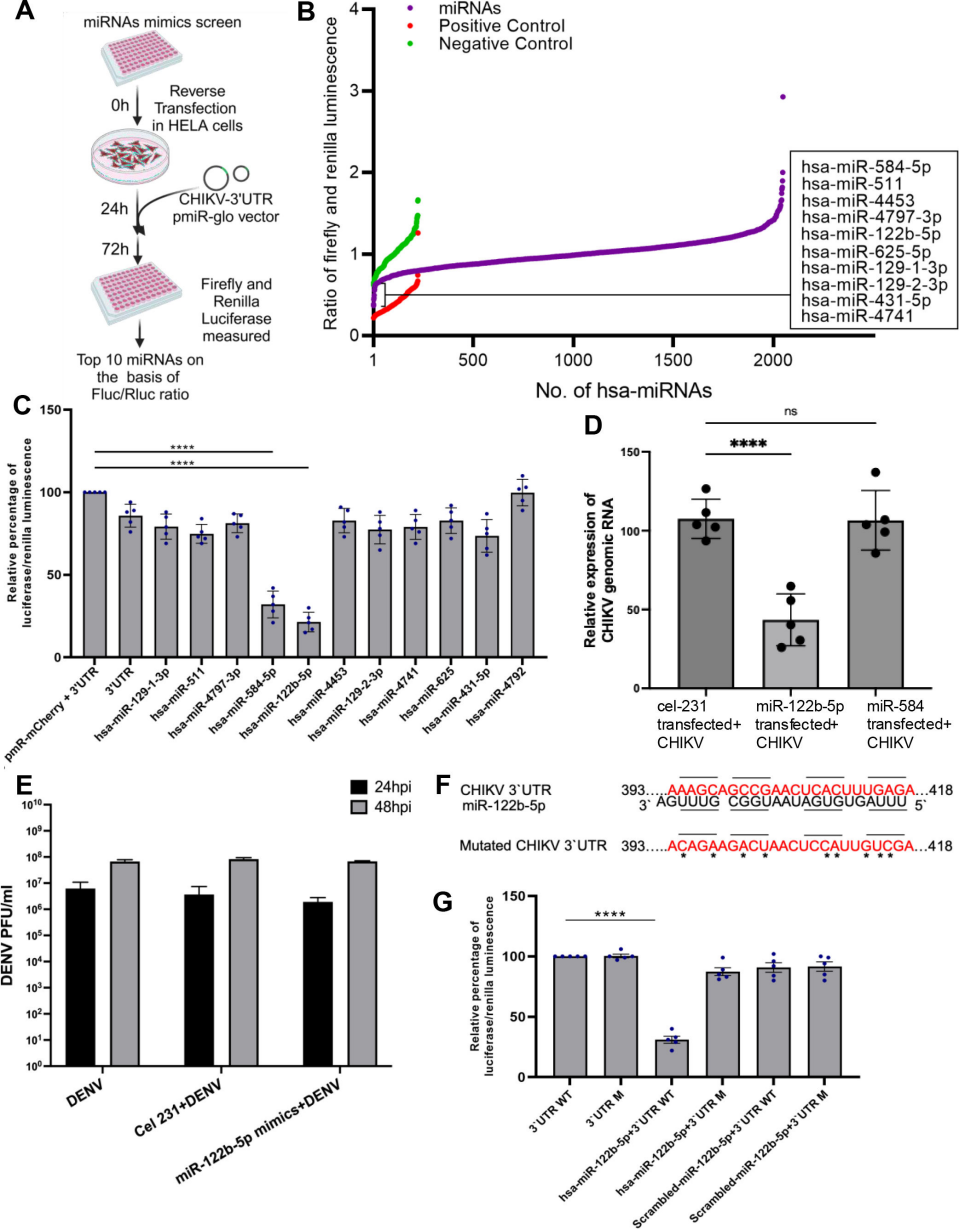
miRNA-122b-5p directly binds to CHIKV 3′-UTR. (**A**) The experimental design (created with biorender.com) of high-throughput screening protocol of human miRNAs mimics with CHIKV 3′UTR. Human miRNAs mimic library was reverse transfected into HeLa cells. Twenty-four hours post-transfection, cells were transfected with cloned CHIKV-3′UTR pmirGLO vector followed by measurement of Firefly and Renilla luciferase. (**B**) The graph shows the ratio of Firefly luciferase and Renilla luminescence of CHIKV 3′-UTR with the library of miRNA mimics (shown in green). miRNAs highlighted in blue are positive control, and those in yellow are negative controls. High-throughput luminescence screening was performed in HELA cells. (**C**) Luciferase assay of human miRNAs targeting the CHIKV 3′-UTR. miRNAs were cloned in pmR-cherry vector, while CHIKV 3′-UTR was cloned in pmiR-GLO vector. Empty pmR-mcherry with CHIKV 3′-UTR was used as a negative control. Data are expressed as mean ± SD, ****P < 0.0001. Bar represents the relative percentage of luciferase/Renilla luminescence for each miRNA tested. Bars are colored gray with individual data points represented as blue dots. (**D**) Relative CHIKV genomic RNA expression in HEK293T cells 24 hpi post-infection following miRNA mimic transfection. Cells were transfected with cel-231 (mock), miR-584-5p, or miR-122b-5p mimics and infected with CHIKV (MOI = 1). CHIKV RNA levels were measured using qRT-PCR and calculated using 100 × 2^-ΔCt^. Bars represent mean ± SD of five biological replicates, with individual values shown. One-way ANOVA followed by Dunnett’s multiple comparisons test was performed using cel-231 as the reference. ****P < 0.0001; ns = not significant. (**E**) A bar graph represents the plaque assay of DENV-infected Vero cells under different conditions: DENV, DENV infected with cel-231 (mock mimics) transfected, and DENV infected with mimic-122b-5p transfected at different time points, 24 hpi and 48 hpi. Data were analyzed using two-way ANOVA between DENV (control) and miR-122b-5p overexpressed with infected condition. Data are expressed as mean ± SD. (**F**) Nucleotide sequence of miR-122b-5p and CHIKV 3′-UTR binding sequence underlined. The sequence of the 3′-UTR of CHIKV recognized by miR-122b-5p and the 3′-UTR mutated sequence (stars) used in the target validation assay is shown. (**G**). Luciferase assay of CHIKV 3′-UTR wild-type (3′UTR WT) and CHIKV 3′-UTR mutant type (3′UTR M) with miR-122b-5p mimics in HEK cells. Scrambled miRNA was used as a negative control. Results were plotted as mean Firefly Luc activity (relative light units [RLUs]) standardized to control Renilla Luc activity ± SD, ****P < 0.0001. Bars represent the relative percentage of luciferase/Renilla luminescence for different 3′-UTR conditions. Bars are colored gray with individual data points as blue dots. Wild-type (WT) 3′-UTR and mutated 3′-UTR conditions are indicated. The experiments were repeated more than three times, and each experiment included at least three treatments.

To investigate the direct binding of human miRNAs to the 3′ untranslated region (3′UTR) of CHIKV, we conducted a luminescence-based high-throughput screening that utilized a library of miRIDIAN miRNA mimics obtained from miRBase release 19.0. miRIDIAN mimic negative controls 2 and 4 were used (negative controls), while two siRNAs specifically designed to target the CHIKV 3′-UTR served as positive controls. The FLuc/RLuc ratio was used as a stringent criterion for each sample, including miRNA mimics, positive controls, and negative controls. Only miRNAs with values falling within the range of positive controls were considered. The experimental design is presented in Fig. 1A. Through this screening, 10 miRNAs directly bound to the CHIKV 3′UTR were identified (Fig. 1B). To further investigate the binding efficiency of these 10 miRNAs with CHIKV 3′UTR, we performed a Dual-Glo luciferase assay using the pmR-mCherry and pmirGLO system. hsa-miR-4792, which was predicted to not target the CHIKV 3′-UTR, was used as negative control. Among the 10 miRNAs, miR-122b-5p and miR-584-5p exhibited the most significant efficacy in targeting the CHIKV 3′-UTR, with a 70% and 80% reduction (*P*-value < 0.0001) in luminescence, respectively (Fig. 1C).

To assess the functional relevance of miR-122b-5p and miR-584-5p in regulating CHIKV replication, HEK-293T cells were transfected with miRNA mimics for miR-122b-5p, miR-584-5p, or a non-targeting control (cel-231). After 24 h of transfection, cells were infected with CHIKV at an MOI of 1, and viral RNA levels were quantified 24 h post-infection using qRT-PCR. CHIKV RNA expression was calculated as 100 × 2^-ΔCt^. A significant reduction in CHIKV RNA levels was observed in miR-122b-5p-transfected cells compared to mock-transfected controls (cel-231), with an approximately 60% decrease (*P* < 0.0001, Dunnett’s multiple comparisons test). In contrast, miR-584-5p overexpression did not result in a statistically significant change in viral RNA levels (*P* > 0.99), indicating that the antiviral effect was specific to miR-122b-5p (Fig. 1D). To check the specificity of the miR-122b-5p mimic for CHIKV, we transfected the mimic into Vero cells, infected the cells with DENV, and observed no change in DENV viral titer. cel-231 served as a negative control for this experiment (Fig. 1E).

To provide further confirmation that the CHIKV 3′-UTR is indeed a direct target of miR-122b-5p, we utilized the RNAhybrid tool to predict the binding sites of this specific miRNA on the CHIKV 3′-UTR. Subsequently, we introduced mutations in the predicted binding sites of mature miRNA sequence by employing site-directed mutagenesis (Fig. 1F), and the mutants were evaluated using the Dual-Glo luciferase assay. The results of the assay demonstrated that in the presence of the miR-122b-5p pmcherry vector, no reduction in luminescence was observed in the mutated CHIKV 3′-UTR (referred to as 3′-UTR M). Conversely, a significant reduction of approximately 70% was observed in the CHIKV 3′-UTR wild type (referred to as 3′-UTR WT) (Fig. 1G). As an additional control, a scrambled miR-122b-5p sequence was used, which exhibited no reduction when tested with the CHIKV 3′-UTR wild type. Taken together, these results indicate that exogenously transfected miR-122b-5p directly targets the CHIKV 3′-UTR.

### miR-122b-5p regulates CHIKV infection in THP-1 derived macrophages

**Fig 2:**
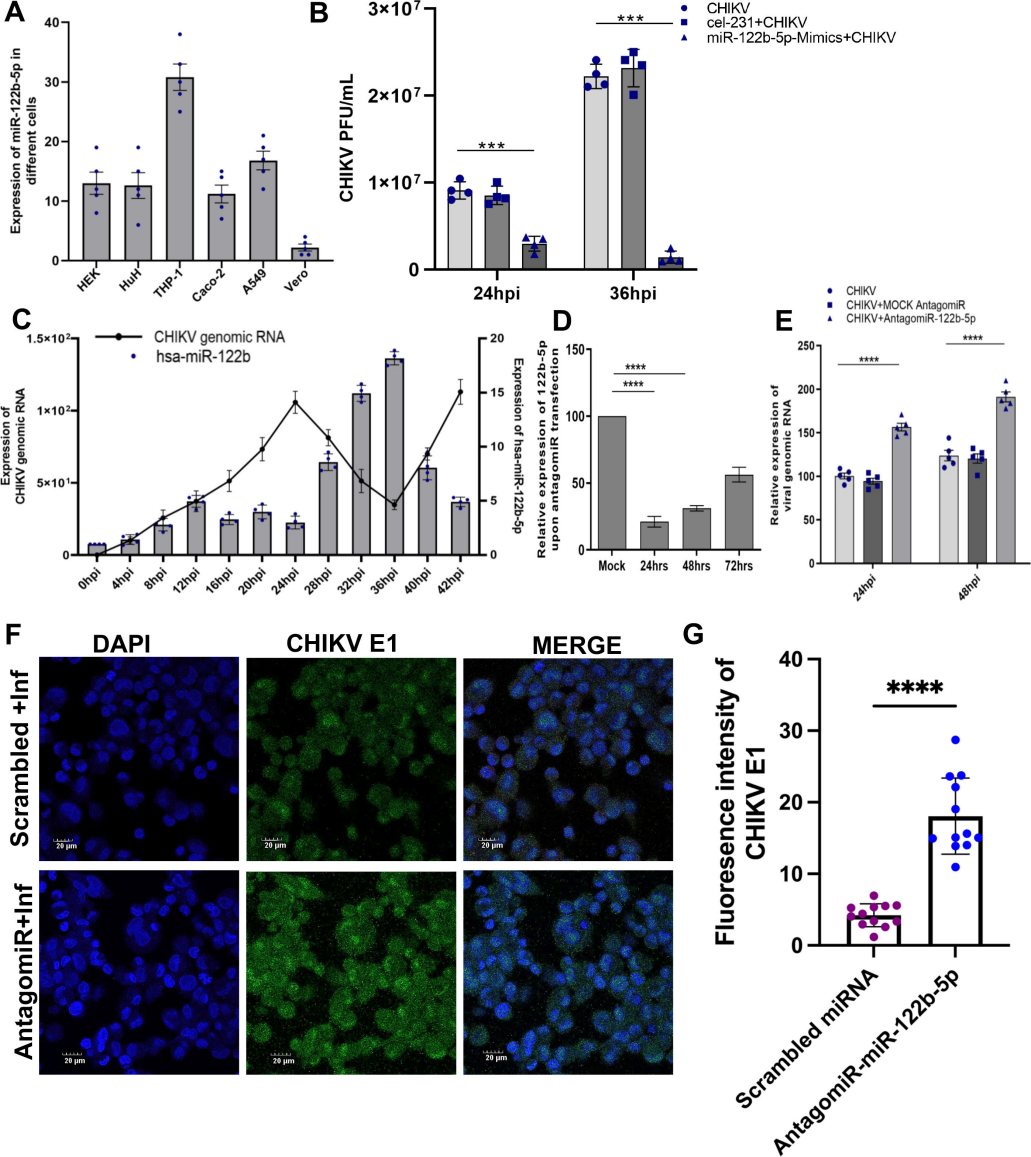
Upregulation of miR-122b-5p in CHIKV-infected macrophages (**A**). The bar represents the expression of miR-122b-5p in different CHIKV-susceptible human cells. The experiments were repeated more than thrice, and statistical significance was determined using Student’s *t*-test (*n* = 4 biological replicates). Bars are gray, and individual data points are shown in blue (**B**). Bars represent plaque assay of CHIKV infected Vero cells under different conditions: CHIKV, CHIKV infected with cel-231 (mock mimics) transfected, and CHIKV infected with mimic-122b-5p transfected at different time points, 24 hpi, and 36 hpi. Individual data conditions are displayed using distinct shapes: blue circles represent CHIKV-infected Vero cells, red squares represent CHIKV infected with cel-231 (mock mimics) transfected conditions, and green triangles represent CHIKV infected with mimic-122b-5p-transfected conditions. Data were analyzed using two-way ANOVA between CHIKV (control), cel-231, and miR-122b-5p overexpressed with infected conditions. Data are expressed as mean ± SD, ****P < 0.0001. (**C**) Expression profiling of viral genomic RNA and miR-122b-5p level at different infection time points in human macrophages (THP-1). Bar represents the expression of miR-122b-5p, and line represents the level of CHIKV genomic RNA (**D**). The bar graph represents the expression level of miR-122b-5p in THP-1 cells, transfected with antagomiR-122b-5p. Transfected cells were harvested at different time points, i.e., 24 h, 48 h, and 72 h. Scrambled miRNA was used as mock antagomiR. (**E**) Bars represent relative expression of CHIKV genomic RNA in human macrophages under different conditions (CHIKV infection, CHIKV infection with scrambled miRNA [mock antagomiR], and CHIKV infection with antagomiR-122b-5p and at different time points, 24 hpi, and 48 hpi). Individual data conditions are displayed using distinct shapes: circles represent CHIKV-infected THP1-derived macrophages, squares represent CHIKV infected with mock antagomiR-transfected conditions, and triangles represent CHIKV infected with antagomiR-122b-5p-transfected conditions. Data were analyzed using one-way ANOVA between CHIKV (control) and miR-122b-5p inhibited with infected condition. Data are expressed as mean ± SD, ****P < 0.0001. (**F**) THP-1 cells were transfected with antagomiR-miR-122b-5p and scrambled miRNA (negative control). Twenty-four hours post-transfection, cells were infected with CHIKV (MOI 1). Cells were fixed and stained with anti-CHIKV-E1 (green) antibody. DNA was visualized with DAPI (blue). The experiments were repeated at least thrice, and each experiment included at least three treatments. Data are expressed as mean ± SD, ***P < 0.001 ****P < 0.0001. (**G**) A bar graph displays mean fluorescence intensity of CHIKV E1 in the cytoplasm for scrambled control versus AntagomiR-122b-5p-treated CHIKV-infected cells. Each dot represents an individual cell quantified (*n* ≥ 20 cells per condition). Data are shown as mean ± SD from three independent biological replicates. Statistical significance was assessed using an unpaired two-tailed *t*-test; ****P < 0.0001.

To understand the biological relevance of endogenously expressed miR-122b-5p during CHIKV infection, we first determined the levels of this miRNA in different CHIKV-susceptible cells, such as human hepatocytes (Huh), human embryonic kidney cells (HEK293), human colorectal adenocarcinoma cells (Caco-2), human alveolar basal epithelial cells (A549), and human monocyte-derived macrophages (THP-1). African green monkey–derived Vero cells, which are highly susceptible to CHIKV, were used as a control. miR-122b-5p was endogenously expressed in all human cell types and was particularly abundant in THP-1 macrophages, while it was found to be negligibly expressed in Vero cells (Fig. 2A). To confirm the specificity of miR-122b-5p to human cells and its role in CHIKV infection, we overexpressed miR-122b-5p in Vero cells using mimic-miR-122b-5p transfection. Following transfection, the cells were infected with CHIKV at MOI 1, and viral titers were measured at 24 and 36 h post-infection (hpi). At 24 hpi, overexpression of miR-122b-5p (mimics condition) led to a slight but significant reduction in viral titers compared to CHIKV infection alone (*P* < 0.001). By 36 hpi, the reduction in viral titers in mimics condition was more pronounced, showing a significant decrease compared to both the CHIKV-only and cel-miR-231 control conditions (*P* < 0.0001). No significant difference was observed between the CHIKV-only and cel-miR-231 control conditions at either time point, confirming the specificity of the observed effect (Fig. 2B). Taken together, these observations suggest that miR-122b-5p could inhibit CHIKV infection in macrophages.

Earlier studies have proved that CHIKV persists in macrophages at an extremely low level, which could contribute to the chronicity of infection (28, 29). We hypothesized that miR-122b-5p could be playing a regulatory role in this persistence of infection. As a first step, using THP-1-derived macrophages, we monitored CHIKV intracellular viral RNA levels over time up to 42 hpi. In macrophage cells, genomic viral RNA levels presented a biphasic trend. First, it progressively increased to peak at 24 hpi, then declined until 36 hpi and subsequently rose again at 42 hpi (Fig. 2C). At the same time points, we monitored the levels of endogenously expressed miR-122b-5p. We found that a negative correlation existed between the miRNA and CHIKV RNA titer (Fig. 2C). Furthermore, to validate this negative correlation between the levels of miR-122b-5p and those of CHIKV RNA, we suppressed the miRNA using a miRNA antisense inhibitor (antagomiR) before infecting the THP-1 with CHIKV. An optimal silencing of 80% for the miRNA was achieved at 24 hpi (Fig. 2D). Based on this, infection with CHIKV (MOI 1) was performed at this time point to quantify the intracellular viral RNA titer. When miR-122b-5p was silenced, we observed significant changes in viral titer at both 24 hpi and 48 hpi (less than twofold) compared to CHIKV and CHIKV mock-antagomiR controls at the respective time points (Fig. 2E). Although the changes are small, they could still have biological significance in the context of viral replication dynamics, as even slight increases in viral titer may contribute to enhanced viral spread or host–pathogen interactions over time. Furthermore, confocal imaging in miR-122b-5p-silenced and CHIKV-infected cells showed an increase in fluorescence intensity of CHIKV-E1 protein level compared to scrambled miRNA and CHIKV-infected cells (Fig. 2F and G). These findings confirm that miR-122b-5p indeed negatively regulates CHIKV infection in macrophages.

### Cellular HDAC4 mRNA is a target of miR-122b-5p

**Fig 3:**
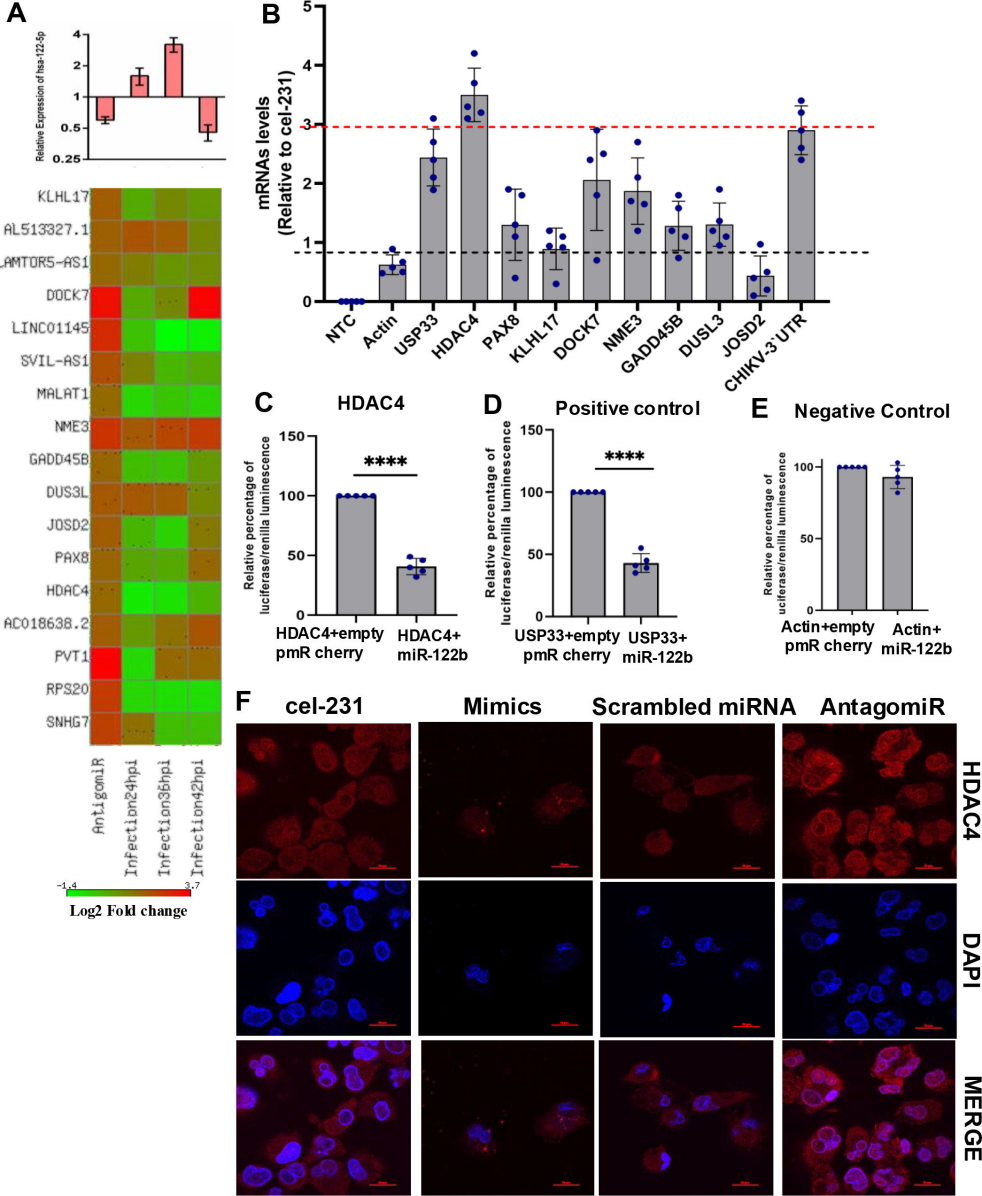
CHIKV induces major changes in the transcriptome of infected macrophages. (**A**) The expression level of miR-122b-5p in different library samples (top panel) and heatmap data show the genes upregulated upon miR-122b-5p inhibition and during CHIKV infection (bottom panel). (**B**) miR-122b-5p targets identification using a biotinylated miRNA pull-down assay in THP-1 cells. Bars represent mRNA levels of different targets in CHIKV infected with overexpressed miR-122b samples normalized to CHIKV infected with cel-231 (mock-mimics) overexpressed conditions. The data were compared to actin mRNA (negative control). No amplification was observed in the no template controls (NTC). The dashed red line shows mRNA level of USP33, while the dashed black line indicates the baseline threshold of actin mRNA. All reactions were performed in triplicate, and data were analyzed using the 2-ΔCt method and expressed as mean ± SD. (**C**) Luciferase assay showing the relative percentage of Firefly/Renilla luminescence for assessing miR-122b-5p regulation of the cellular target HDAC4. HEK293T cells were co-transfected with HDAC4 3′UTR cloned in the pmirGLO vector and either empty pmR-mCherry (control) or pmR-miR-122b-5p expression vector. (**D**) USP33, a validated target of miR-122b-5p, was used as a positive control under identical assay conditions. (**E**) Actin 3′-UTR served as a negative control to confirm the specificity of miR-122b-5p binding. All constructs were cloned in the pmirGLO vector and co-transfected with pmR-miR-122b-5p or empty pmR-mCherry. Firefly luciferase activity was normalized to Renilla luciferase and expressed as a percentage relative to the empty vector control. Data represent mean ± SD from three independent biological replicates. (**F**) THP-1 cells were transfected with miR-122b-5p mimic, cel-231 mimic, antagomiR-122b-5p, or scrambled miRNA. Scrambled miRNA and cel-231 were used as negative controls for antagomiR and mimics, respectively. Subsequently, cells were stained with DAPI (blue) and anti-HDAC4 (red) and subjected to confocal imaging. Scale bar 20 µm. All experiments were repeated at least thrice, and each experiment included at least three treatments.

To further evaluate the role of miR-122b-5p during CHIKV infection, besides directly targeting the CHIKV 3′UTR, we performed a global transcriptome profiling of THP-1-derived macrophages at 24 hpi, 36 hpi, and 42 hpi, following miR-122b-5p inhibition to identify the cellular targets of this miRNA. Also, we quantified miR-122b-5p in the above samples and observed an upregulation of this miRNA at 24 hpi and 36 hpi and downregulation at 42 hpi, while miR-122b-5p expression was reduced to ~40% upon treatment with the antagomiR-122b (Fig. 3A, top panel). Based on *P*-values (*P* ≤ 0.05) and log FC (±1.5), 27 targets (direct or indirect) of miR-122b-5p were found to be regulated in the antagomiR library when compared to the control (Table S1). Out of these, 17 genes were annotated, of which seven were non-coding (this includes lncRNA, antisense RNA, and other RNA genes), while 10 were coding genes (which included protein-coding transcripts, pseudogenes, and ribosomal proteins; Fig. 3A, bottom panel). We excluded the ribosomal proteins, pseudogenes, and selected eight targets for further validation during CHIKV infection (HDAC4, PAX8, KLHL17, DOCK7, NME3, GADD45B, DUSL3, and JOSD2). The expression profile of these eight genes during CHIKV infection is shown in Fig. S1A.

The eight selected targets of miR-122b-5p were further assessed for their direct binding to miR-122b-5p using biotinylated miRNA pull-down assays and luciferase assays. USP33 was reported to be a target of miR-122b-5p (30) and thus was used as a positive control in both cases, while actin served as the negative control in both the assays. CHIKV 3′-UTR was also assessed to further validate its binding to the miRNA. In the biotinylated pull-down assay, eluted RNA was processed, and the mRNA levels of target genes were quantified using qRT-PCR. Biotinylated cel-231 served as a negative control, while actin mRNA was used as a non-target control to assess specificity. Minimal enrichment of actin mRNA confirmed the specificity of miR-122b-5p binding, while no amplification in no-template control (NTC) reactions validated the accuracy of the assay. Based on thresholds established using actin and USP33 mRNA, the results indicated that HDAC4, DOCK7, and the CHIKV 3′-UTR were the most enriched targets in biotinylated miR-122b-5p pull-down elutes, suggesting direct binding to the miRNA. Notably, the mRNA levels of HDAC4 and CHIKV 3′-UTR were >2.5-fold and twofold higher, respectively, compared to the positive control USP33 mRNA (Fig. 3B). In the case of the luciferase assay, HDAC4 showed a 70% reduction in luciferase/Renilla luminescence, while the positive control USP33 showed a reduction of ~60% and actin did not show any reduction in its luminescence (Fig. 3C through E). The reduction in luminescence of the other targets ranged from ~36% to ~55% (Fig. S1B). Interestingly, miR-122b-5p seems to have a counteractive role through its inhibitory effect on HDAC4. This possibility was further supported by our observation of a marked reduction in HDAC4 in THP-1 macrophages when transfected with miR-122b-5p compared to cel-231-transfected cells (Fig. 3F). Correspondingly, the use of antagomiR-miR-122b-5p led to a reverse effect (Fig. 3F), with a scrambled miRNA serving as a control in the experiment. Taken together, the results identify HDAC4 to be a cellular target of miR-122b-5p that the miRNA negatively regulates.

### HDAC4 regulates CHIKV infection dynamics

**Fig 4:**
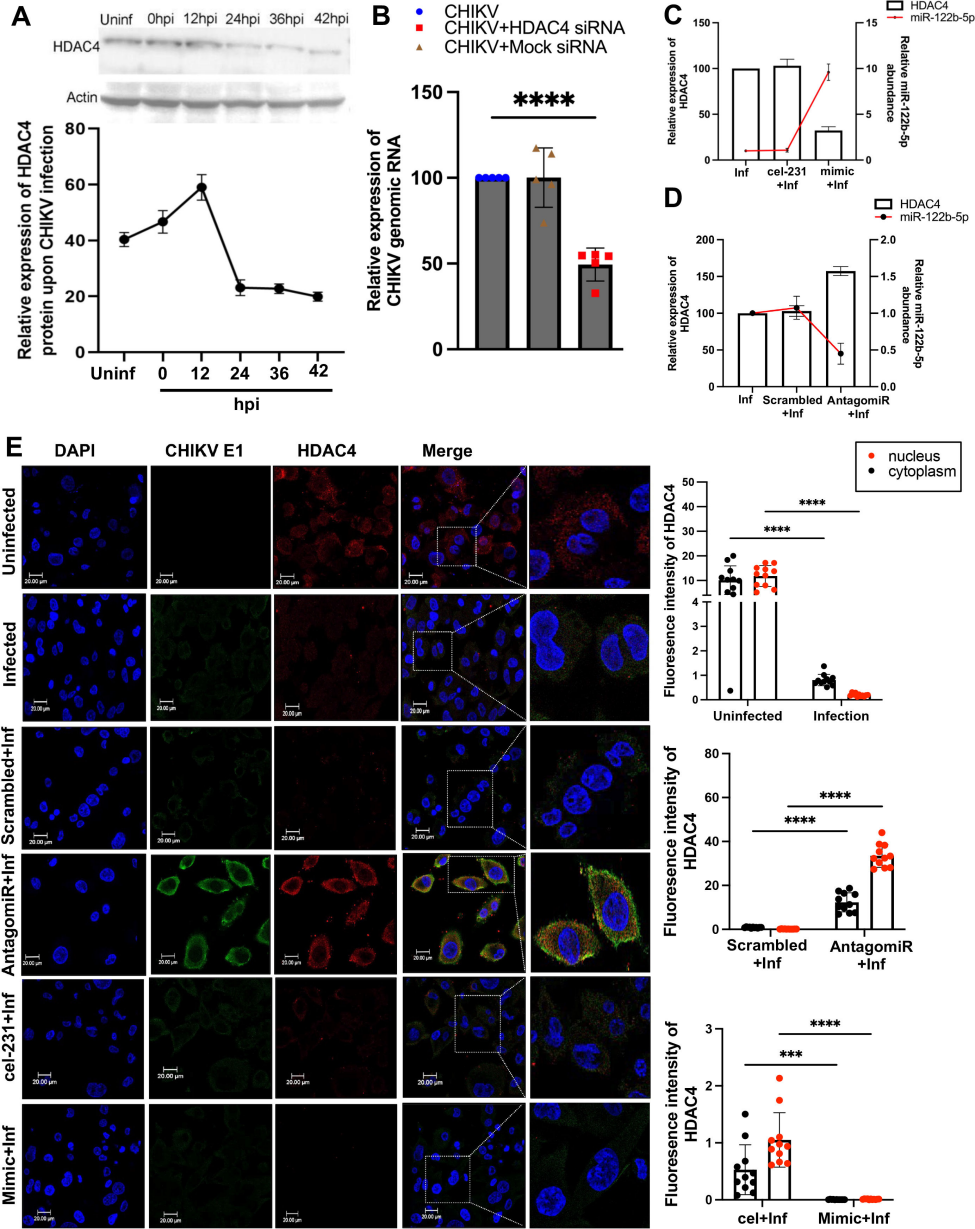
Role of HDAC4 in CHIKV infection (**A**). HDAC4 protein levels at various times after CHIKV infection. The line graph illustrates the relative expression levels of HDAC4 under different experimental conditions (**B**). Relative expression of CHIKV genomic RNA in PMA-activated THP-1-derived macrophages at 12 hpi under different transfection conditions: CHIKV infection alone, CHIKV infection with mock siRNA, and CHIKV infection with HDAC4 siRNA. Each condition shows mean values (gray bars) ± SD from four biological replicates (individual data points: blue circles for CHIKV alone, brown triangles for mock siRNA, and red squares for HDAC4 siRNA). Data were analyzed using the 2^–ΔΔCt^ method and normalized to GAPDH. Statistical analysis was performed using one-way ANOVA with Dunnett’s multiple comparison test, with the CHIKV infection group, ****P < 0.0001. (**C**) Relative expression of HDAC4 and miR-122b-5p in PMA-activated THP-1-derived macrophages at 24 hpi under different transfection conditions: CHIKV infection alone, CHIKV infection with cel-231, and CHIKV infection with mimics-122b-5p. Bars represent HDAC4 transcript levels; the red line represents miR-122b-5p expression. (**D**) Relative expression of HDAC4 and miR-122b-5p in PMA-activated THP-1-derived macrophages at 24 hpi under different transfection conditions: CHIKV infection alone, CHIKV infection with scrambled miRNA, and CHIKV infection with AntagomiR-122b-5p. Bars indicate HDAC4 levels; the red line indicates miR-122b-5p expression. Each condition shows mean values (gray bars) ± SD from four biological replicates. Data represent mean ± SD from three independent biological replicates. Statistical significance was assessed using one-way ANOVA followed by Dunnett’s post hoc test (*P < 0.05, **P < 0.01, ***P < 0.001, ****P < 0.0001). (**E**) Immunofluorescence staining and quantification of HDAC4 and CHIKV E1 in PMA-differentiated THP-1 macrophages under miR-122b-5p modulation. Representative confocal images display DAPI (blue), CHIKV E1 (green), and HDAC4 (red) signals across the following conditions: Uninfected, CHIKV-infected, scrambled + infection, AntagomiR-122b-5*P* + infection, cel-231 + infection, and miR-122b-5p mimic + infection. Full-field scale bars = 20  µm; zoomed-in panels = 10  µm. Bar plots show quantification of HDAC4 fluorescence intensity in the nucleus (red dots) and cytoplasm (black dots) across the indicated conditions. Data are presented as mean ± SD from three independent biological replicates (*n* ≥ 25 cells per group). Statistical significance was determined by one-way ANOVA followed by Dunnett’s post hoc test. *P < 0.05, **P < 0.01, ***P < 0.001, ****P < 0.0001.

Histone deacetylase 4 (HDAC4), a member of the Class II HDACs, has been recognized as a key player in the regulation of immune responses across a wide spectrum of immune cells (31–33). To further explore the implications of HDAC4 in the context of CHIKV-infected macrophages, we profiled the expression of HDAC4 in THP-1 macrophages following CHIKV infection at 12, 24, 36, and 42 hpi. We observed that HDAC4 expression peaked at 12 hpi, after which a significant decrease in its expression was observed post 24 hpi and plateaued at low expression levels until 42 hpi (Fig. 4A). To elucidate if HDAC4 was involved in regulating CHIKV infection, we silenced the gene and evaluated the impact of its silencing on CHIKV infection at 12 hpi. We observed a significant reduction (*P*-value < 0.001) in viral genomic RNA at 12 hpi in the HDAC4 knockdown cells (Fig. 4B). To establish the regulatory connection between miR-122b-5p and HDAC4, we quantified their expression under varying experimental conditions. Overexpression of miR-122b-5p (mimic + infection) led to a significant increase in miRNA levels accompanied by a concurrent reduction in HDAC4 expression (Fig. 4C). Conversely, inhibition of miR-122b-5p (AntagomiR + infection) resulted in decreased miRNA levels and a marked upregulation of HDAC4 (Fig. 4D), reinforcing an inverse regulatory relationship. These patterns substantiate the regulatory role of miR-122b-5p in modulating HDAC4 expression during CHIKV infection.

To further validate the inverse regulation of HDAC4 by miR-122b-5p at the cellular level, we performed immunofluorescence imaging in PMA-differentiated THP-1 macrophages under miRNA overexpression and silencing conditions. Confocal analysis revealed that in uninfected cells, HDAC4 was predominantly localized to the cytoplasm. Upon CHIKV infection, HDAC4 levels were significantly reduced in both nuclear and cytoplasmic compartments, with a more pronounced decline in the cytoplasm (Fig. 4E, rows: uninf- inf). AntagomiR-122b-5p treatment effectively restored HDAC4 cytoplasmic intensity to levels significantly higher than those observed in scrambled controls, indicating that miR-122b-5p inhibition alleviates HDAC4 repression (Fig. 4E, rows: scrambled + inf and antagomiR + inf). In contrast, overexpression of miR-122b-5p markedly suppressed HDAC4 expression in both compartments relative to the cel-231 control (Fig. 4E, rows: cel-231 + inf and mimics + inf), supporting the hypothesis that miR-122b-5p facilitates HDAC4 degradation or inhibition. Collectively, these findings confirm HDAC4 as a downstream target of miR-122b-5p and highlight miR-122b-5p’s regulatory role in modulating both HDAC4 expression and its intracellular trafficking during CHIKV infection. This regulatory axis underscores the potential of miR-122b-5p as a critical modulator of HDAC4-mediated pathways, influencing both viral replication dynamics and host cellular responses.

### HDAC4 and miR-122b-5p mediate regulation of cytokines and interferon-stimulated genes upon CHIKV infection

**Fig 5:**
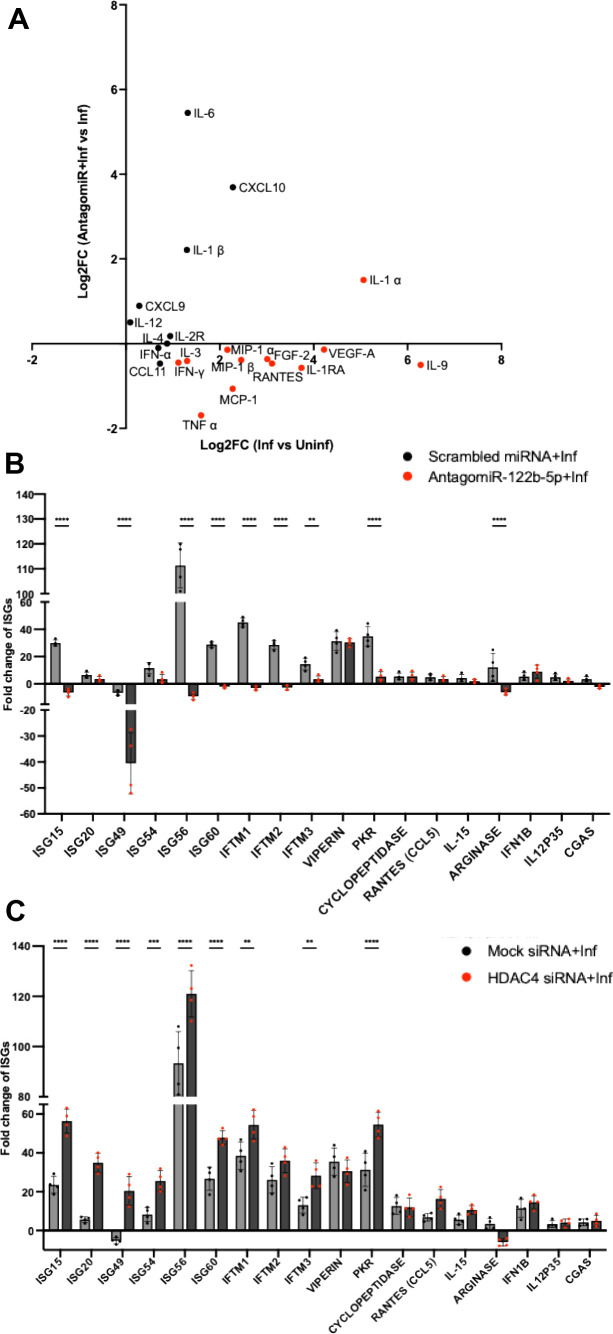
HDAC4 and miR-122b-5p regulate cytokines and ISGs upon CHIKV infection in THP1-derived macrophages. (**A**) Scatter plot depicting Log₂ fold changes (Log₂FC) in cytokine expression in CHIKV-infected THP-1 cells at 24 h post-infection (x-axis: CHIKV-infected vs. uninfected) and AntagomiR-122b-5p-treated CHIKV-infected cells vs. CHIKV-infected cells (y-axis). Each dot represents an individual cytokine. Red points highlight cytokines that were significantly induced by CHIKV infection but were downregulated upon miR-122b-5p inhibition, indicating potential miRNA-mediated regulation. Data are shown as mean Log₂FC from three independent biological replicates. A paired two-tailed *t*-test revealed a significant overall reduction in cytokine induction following AntagomiR treatment (*P* = 0.0034). (**B**) Fold change of ISG expression upon miR-122b-5p silencing at 24 hpi. This time point was chosen because AntagomiR transfection remains stable until 24 hpi, after which its effect diminishes due to degradation. Scrambled miRNA with infected cells was used as a negative control. Data were analyzed using the 2^−ΔΔCt^ method and expressed as mean ± SD. ***P < 0.001, **P < 0.0001. Statistical significance was calculated using two-way ANOVA. (**C**) Fold change of ISG expression upon HDAC4 silencing at 12 hpi. Transient HDAC4 siRNA transfection achieves peak knockdown efficiency at 12 hpi but shows reduced efficacy at 24 hpi, likely due to siRNA degradation. Therefore, 12 hpi was selected as the optimal time point for assessing HDAC4-mediated effects on ISG expression. Mock siRNA with infected cells was used as a negative control. Data were analyzed using the 2^−ΔΔCt^ method and expressed as mean ± SD. ***P < 0.001, **P < 0.0001. Statistical significance was calculated using two-way ANOVA.

Following the observed correlation between miR-122b-5p and its target expressions during CHIKV infection, we sought to elucidate the potential mechanism underlying this association. Given that macrophages release inflammatory mediators upon CHIKV infection (34–36), we conducted a quantitative multiplex cytokine profiling using a 35-plex cytokine/chemokine human panel in uninfected, CHIKV-infected, and miR-122b-5p-silenced CHIKV-infected THP-1 cells. Cytokine expression was analyzed by calculating Log2 fold changes (Log2FC) across two comparisons: infected versus uninfected (Inf vs. Uninf) and miR-122b-5p antagomiR-treated infected versus untreated infected (antagomiR + Inf vs. Inf) conditions (Table S2). A scatter plot was generated, plotting the Log2FC of Inf vs. Uninf (x-axis) against the Log2FC of antagomiR + Inf vs. Inf (y-axis) (Fig. 5A). Cytokines significantly upregulated during CHIKV infection but reversed upon antagomiR treatment were highlighted in red. These included IL-1α, FGF-2, RANTES (CCL5), EOTAXIN (CCL11), MCP-1 (CCL2), VEGF-A, IFN-γ, IL-9, IL-1RA, and TNF-α, indicating miR-122b-5p-dependent regulation. Previous studies have demonstrated that pro-inflammatory cytokines and chemokines play a pivotal role in regulating interferon-stimulated genes (ISGs) via the JAK/STAT pathway during viral infections, thereby triggering antiviral responses (37). The observed modulation of these cytokines upon antagomiR treatment indicates a complex regulatory network involving miR-122b-5p and HDAC4 that affects both cytokine production and ISG expression.

To further explore this regulatory axis, we performed qPCR analysis of ISG expression under infected, AntagomiR-treated infected, and HDAC4-silenced infected conditions. The ISGs that exhibited significant regulation upon CHIKV infection are shown in Fig. S2A. Since miR-122b-5p silencing increases CHIKV replication (as observed in Fig. 2E), we quantified CHIKV RNA levels in the same samples used for ISG expression analysis and normalized viral burden before analyzing ISG expression. This ensured that observed ISG expression changes were not solely due to altered viral replication kinetics, but rather a direct effect of miR-122b-5p regulation. We found that a majority of ISGs, including ISG15, ISG49, ISG56, ISG60, IFITM1, IFITM2, IFITM3, Arginase, and PKR, exhibited significant downregulation (*P* < 0.0001, 0.001) compared to cells transfected with scrambled miRNA and subsequently infected with CHIKV (negative controls) (Fig. 5B). Similarly, HDAC4 silencing—after normalization for CHIKV RNA levels—led to a marked upregulation of several ISGs, including ISG15, ISG20, ISG49, ISG54, ISG56, ISG60, IFITM1, IFITM3, and PKR (*P* < 0.0001, 0.001), relative to mock siRNA-transfected CHIKV-infected cells (Fig. 5C), supporting a repressive role of HDAC4 on ISG expression.

To further uncover possible mechanistic links, we performed STRING-based correlation network analysis, revealing that most modulated cytokines and ISGs are directly connected to IRF3, IRF7, and IRF9 (Fig. S2B). However, literature analysis identified a direct interaction between HDAC4 and IRF3, but not with IRF7 or IRF9. Previous studies have demonstrated that HDAC4 suppresses IRF3 phosphorylation, thereby attenuating the antiviral response (31). Collectively, these findings reveal a coordinated regulatory network wherein miR-122b-5p and HDAC4 jointly suppress interferon-stimulated gene expression and cytokine production during CHIKV infection, probably through modulation of IRF3-dependent pathways.

### miR-122b-5p indirectly regulates trafficking of IRF3 in CHIKV-infected macrophages

Type I IFN system is a key antiviral pathway activated upon CHIKV infection, primarily via recognition of viral dsRNA by cellular toll-like receptor 3 (TLR) 3, leading to IRF3 phosphorylation by the kinases TBK1 and IKKε (36, 38, 39). Phosphorylated IRF3 dimerizes—principally through Ser-386 and Ser-396 phosphorylation—and translocates to the nucleus, where it induces interferon-stimulated genes (ISGs). HDAC4 has been previously shown to suppress IRF3 activation by interacting with TBK1/IKKε, thereby inhibiting IRF3 phosphorylation (40, 41). HDAC4 is known to play a crucial role in decreasing type I IFN production, as it gets phosphorylated by TBK1/IKKε, thus preventing IRF3 phosphorylation (31). Earlier research highlights the pivotal role of dimerization in IRF3 activity to study its functionality (42, 43).

**Fig 6:**
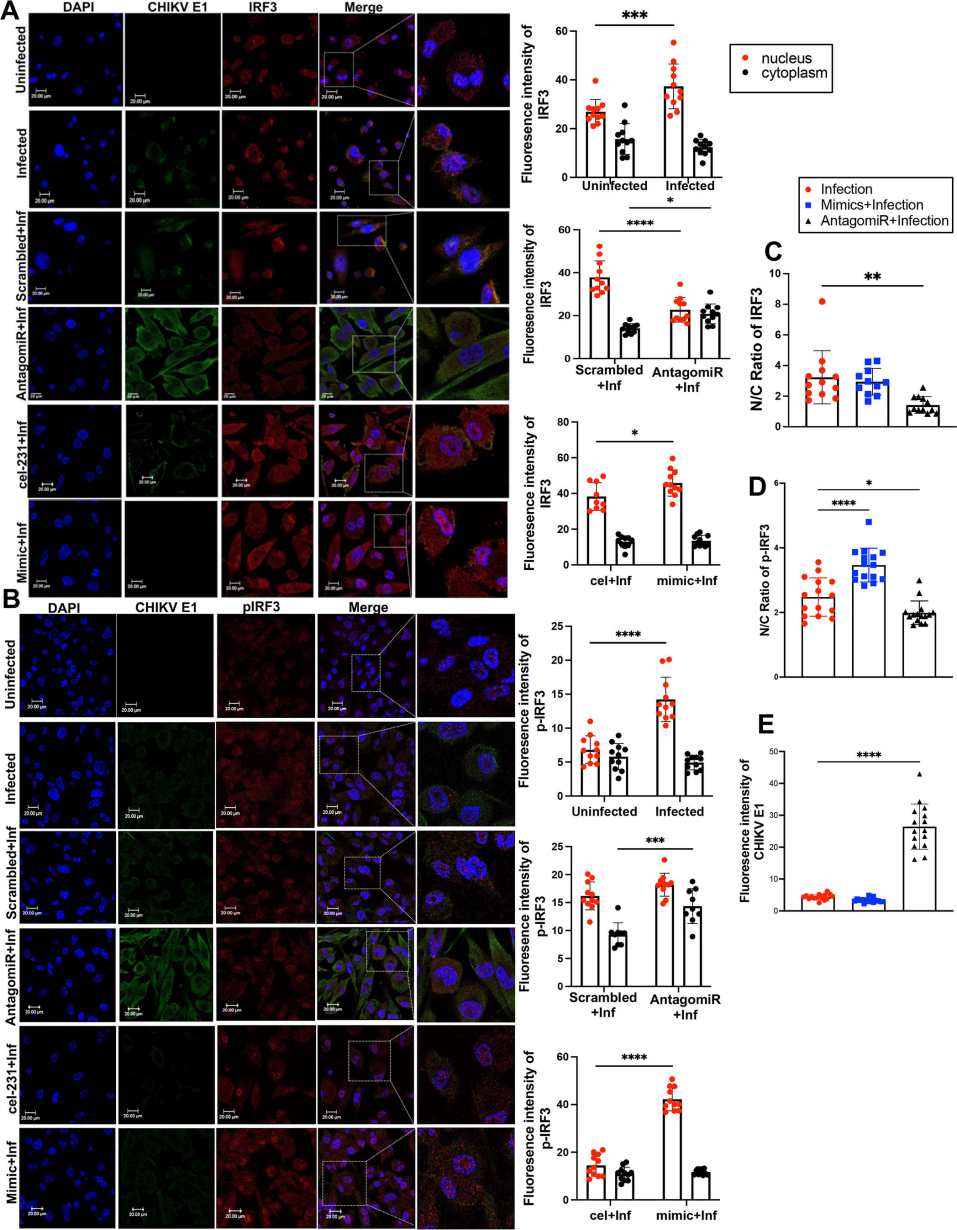
Indirect regulation of IRF3 by miR-122b-5p in CHIKV-infected cells. (**A**) Immunofluorescence staining and quantification of IRF3 and CHIKV E1 in PMA-differentiated THP-1 macrophages under miR-122b-5p modulation. THP-1 cells were infected with CHIKV (MOI 10). Twenty-four hours post-infection, cells were washed with PBS followed by fixation with 4%PFA and permeabilization. Further cells were probed with CHIKV-E1 (green) and anti-IRF3 (red), and the nucleus was stained with DAPI (blue). The conditions are uninfected, CHIKV-infected, scrambled + infection, AntagomiR-122b-5*P* + infection, cel-231 + infection, and miR-122b-5p mimic + infection. Full-field scale bars = 20  µm; zoomed-in panels = 10  µm. Bar plots show quantification of IRF3 fluorescence intensity in the nucleus (red dots) and cytoplasm (black dots) across the indicated conditions. Data are presented as mean ± SD from three independent biological replicates (*n* ≥ 25 cells per group). Statistical significance was determined by one-way ANOVA followed by Dunnett’s post hoc test. *P < 0.05, **P < 0.01, ***P < 0.001, ****P < 0.0001. (**B**) Immunofluorescence staining and fluorescence intensity analysis of phosphorylated IRF3 (p-IRF3) and CHIKV E1 in PMA-differentiated THP-1 macrophages under various miR-122b-5p modulation conditions. Representative confocal micrographs show DAPI (blue), CHIKV E1 (green), and p-IRF3 (red) staining under the following conditions: uninfected, CHIKV-infected, scrambled + infection, AntagomiR-122b-5*P* + infection, cel-231 + infection, and miR-122b-5p mimic + infection. Full-field images: scale bar = 20  µm; zoomed-in regions: scale bar = 10  µm. Bar plots on the right depict quantification of p-IRF3 fluorescence intensity in the nucleus (red dots) and cytoplasm (black dots) for each condition. Data are shown as mean ± SD from three independent biological replicates (*n* ≥ 25 cells per group). Statistical comparisons were performed using one-way ANOVA followed by Dunnett’s post hoc test. *P < 0.05, **P < 0.01, ***P < 0.001, ****P < 0.0001. (**C**) Nuclear-to-cytoplasmic (N/C) ratio of total IRF3. (**D**) N/C ratio of phosphorylated IRF3 (p-IRF3). (**E**) Cytoplasmic intensity of CHIKV-E1 protein as a measure of viral burden in infection, mimic + infection and AntagomiR + infection. Data represent mean ± SD from individual cells (*n* > 25 per group). Statistical analysis was performed using one-way ANOVA followed by Dunnett’s multiple comparisons test using CHIKV infection as the reference. ****P < 0.0001; *P < 0.05; ns, not significant.

To investigate whether miR-122b-5p influences this pathway by modulating HDAC4, we evaluated IRF3’s localization and activation in CHIKV-infected PMA-activated THP-1 macrophages using confocal microscopy and quantitative fluorescence intensity analysis. In uninfected cells, IRF3 expression was low and was predominantly cytoplasmic (Fig. 6A, row: uninf). Upon CHIKV infection, IRF3 expression markedly increased, with significant nuclear accumulation (*P* < 0.001) indicative of activation and nuclear translocation (Fig. 6A, row: inf). Inhibition of miR-122b-5p using an AntagomiR reduced nuclear IRF3 signal and increased cytoplasmic retention compared to scrambled controls (Fig. 6A, rows: scrambled + inf and antagomiR + inf), suggesting impaired nuclear import of IRF3. Conversely, overexpression of miR-122b-5p via mimic transfection enhanced nuclear IRF3 accumulation relative to the cel-231 + infection condition (Fig. 6A, rows: cel-231 + inf and mimics + inf). Quantitative analysis confirmed that mimic-treated cells exhibited increased nuclear IRF3 intensity without a corresponding rise in cytoplasmic signal, indicating enhanced nuclear retention of IRF3 under miR-122b-5p upregulation. We next examined IRF3 phosphorylation using a phospho-specific IRF3 (p-IRF3) antibody. CHIKV infection significantly increased nuclear p-IRF3 levels relative to uninfected cells (*P* < 0.01) (Fig. 6B, row: inf), with minimal changes in cytoplasmic intensity, consistent with activation-induced translocation. AntagomiR treatment resulted in reduced nuclear p-IRF3 and modest cytoplasmic accumulation (Fig. 6B, rows: scrambled + inf and antagomiR + inf), while miR-122b-5p overexpression markedly enhanced nuclear p-IRF3 signal (Fig. 6B, rows: cel-231 + inf and mimics + inf).

To quantitatively capture the subcellular distribution, we calculated the nuclear-to-cytoplasmic (N/C) fluorescence intensity ratios of IRF3 and p-IRF3. AntagomiR treatment significantly reduced the N/C ratio of total IRF3 relative to CHIKV infection alone (*P* < 0.01), while miR-122b-5p mimic increased it modestly (Fig. 6C) indicating cytoplasmic retention of IRF3. A similar trend was observed for p-IRF3: mimic transfection elevated the N/C ratio (*P* < 0.0001), and AntagomiR treatment decreased it (*P* < 0.05) compared to CHIKV infection alone (Fig. 6D). Importantly, changes in IRF3 and p-IRF3 localization inversely correlated with CHIKV-E1 protein levels in the infected cells. CHIKV-E1 fluorescence intensity was highest in AntagomiR-treated cells and lowest in mimic-treated cells (*P* < 0.0001), indicating that reduced IRF3 activation and nuclear localization are associated with enhanced viral infection (Fig. 6E). Together, these findings support a model in which miR-122b-5p contributes to enhanced IRF3 activation and nuclear translocation during CHIKV infection, potentially through modulation of HDAC4 levels, thereby influencing type I IFN signaling.

## DISCUSSION

Previous research has established that miRNAs are pivotal modulators of host–pathogen dynamics, functioning either through direct interactions with viral RNAs or by regulating host gene expression (44, 45) (46, 47). In alphaviruses, including CHIKV, such miRNA-mediated interactions have been reported in both vertebrate and invertebrate hosts (48, 49). In the present study, through a high-throughput screening of a human miRNA mimic library followed by individual validations during infection, we identified miR-122b-5p as a potent negative regulator of CHIKV replication. Despite miR-584-5p also exhibiting binding affinity for the CHIKV 3′-UTR, it did not affect viral replication, suggesting that binding alone is not predictive of antiviral activity. This aligns with existing literature on the context-dependent nature of miRNA functionality, where certain miRNA–viral RNA interactions may stabilize rather than destabilize viral genomes (50–53). The miR-122 cluster is a well-studied miRNA cluster due to the association of miR-122a-5p with cholesterol metabolism and hepatocellular carcinoma, as well as its significant role in promoting hepatitis C virus (HCV) replication (50). This miRNA is encoded within the RNA-positive strand in chromosome 18q21.31 and is known to cluster with miR-3591, which is present within the negative strand at the same position. At the time of our experiments, the miRNA was designated as miR-3951; however, the most recent miRBASE version has renamed miR-3591 as miR-122b-5p (miRbase 22.1). While miR-122a has been well studied for its association with HCV, little is known about miR-122b-5p, apart from a report on its possible role in periodontitis (51). Interestingly, despite the phenotype associated with miR-122b-5p in our study, we observed limited canonical base pairing between the miRNA and the CHIKV 3′-UTR, either within the seed sequence or the body of the mRNA. This finding aligns with prior studies suggesting that miRNAs can exert effects through non-canonical base pairing or indirect mechanisms (52–55). In the context of CHIKV, we hypothesize that miR-122b-5p may interact with secondary structures within the viral 3′-UTR to facilitate non-canonical interactions, highlighting a complex mechanism. Our study is the first to demonstrate the role of this miRNA in viral infection and in eliciting an antiviral response.

The first indication is the negative correlation of miRNA expression and CHIKV infection kinetics in macrophages. During the early stages of CHIKV infection (4–16 hpi), miR-122b-5p levels remained relatively stable, while CHIKV RNA synthesis steadily increased. This suggests that miR-122b-5p may function as part of a feedback mechanism that attenuates viral replication after the initial stages of infection. Although miR-122b-5p levels plateaued between 28 hpi and 40 hpi, divergent viral RNA kinetics during this period suggest dynamic regulation or functional modulation of the miRNA, possibly through miRNA turnover, RNA-binding proteins, or competition with other molecular interactions (56). Furthermore, the study on the expression profiling of all mammalian miRNAs revealed that miR-122b-5p is expressed at varying levels across the various tissues, emphasizing the important regulatory role of this miRNA (57). The same study also reported that this miRNA expressed at extremely low levels in mice (57) compared to its expression in human cells. This indirectly emphasized that we will not be able to employ murine models to study this miRNA in greater detail.

Importantly, we identify a potential secondary role of miR-122b-5p in possibly modulating IRF3 nuclear localization, which may be associated with modulations in the miRNA cellular target, HDAC4 levels. However, the precise mechanistic pathway remains to be established. HDAC4 is a class IIa histone deacetylase previously implicated in suppressing type I IFN signaling, largely through inhibition of IRF3 phosphorylation and activation (31, 58–60). IRF3, a member of the IFN regulatory transcription factor (IRF) family, is well known to be a key player in the activation of early innate immune response upon viral infection (38, 61). We observed that silencing HDAC4 enhanced expression of pro-inflammatory cytokines, such as IL-6, TNF-α, RANTES, MIP-1α/β, MCP-1, IL-1RA, and IL-17F—cytokines that are central to antiviral immunity and are largely downstream of IRF3 activation (62–65). IRF3, when activated through phosphorylation by TBK1/IKKε, dimerizes and translocates into the nucleus to initiate transcription of interferon-stimulated genes (ISGs). This cascade plays a pivotal role in early viral sensing and clearance. Indeed, studies have shown that mice deficient in IFN-β exhibit exacerbated CHIKV pathology, further underscoring the relevance of the IRF3–IFN-β axis in controlling infection and inflammation (64).

We further analyzed this phosphorylation status of IRF3 by subcellular localization analysis using fluorescence intensity analysis. We observed CHIKV infection alone moderately increased nuclear p-IRF3 levels, reflecting the virus’s inherent ability to activate host antiviral defenses. However, overexpression of miR-122b-5p via mimics substantially enhanced nuclear retention of p-IRF3 and total IRF3, indicating potentiation of the innate immune response. In contrast, antagomiR-mediated inhibition of miR-122b-5p was associated with prominent cytoplasmic retention of IRF3 and p-IRF3, suggesting a possible reduction in phosphorylation and impaired nuclear translocation of IRF3. These changes directly correlated with viral protein (E1) abundance—lowest in mimic-treated cells and highest in antagomiR-treated conditions—further validating the antiviral role of this miRNA.

**Fig 7:**
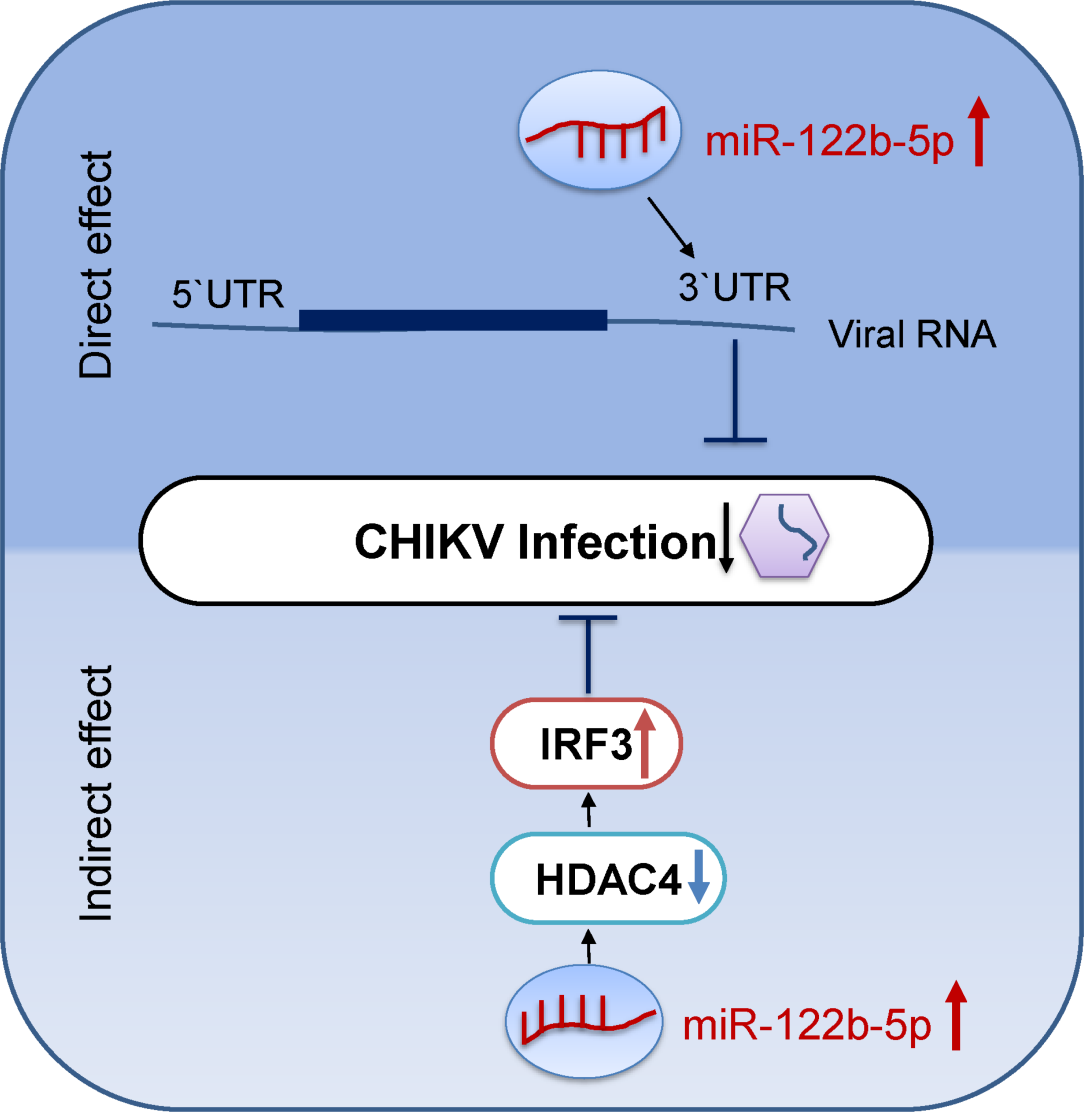
Schematic model illustrating the dual regulatory role of miR-122b-5p in CHIKV infection. The figure depicts both the direct and indirect effects of miR-122b-5p upregulation during Chikungunya virus (CHIKV) infection. Direct effect (top): miR-122b-5p binds to the 3′ untranslated region (UTR) of CHIKV genomic RNA, leading to its translational repression or degradation. Indirect effect (bottom): miR-122b-5p targets the cellular transcript HDAC4, leading to its downregulation. Reduced HDAC4 levels may relieve repression on IRF3, potentially enhancing IRF3 expression and activity. Increased phosphorylated IRF3 levels are associated with an elevated antiviral response, thereby contributing to CHIKV inhibition. This model underscores the dual antiviral role of miR-122b-5p via both viral RNA targeting and host immune signaling modulation.

Overall, these findings establish miR-122b-5p as a dual-action antiviral regulator: It inhibits CHIKV replication by directly targeting the viral 3′-UTR and may also contribute to enhanced host antiviral responses. This latter effect may involve modulation of HDAC4, which in turn might affect IRF3 activation, although this potential connection remains to be clarified through further investigation. (Fig. 7). These findings underscore the importance of spatial protein dynamics and highlight miR-122b-5p as a potential dual-action therapeutic target—suppressing CHIKV replication through both direct genomic binding and indirect modulation of host antiviral pathways.

## MATERIALS AND METHODS

### Cell lines

In this study, THP-1 cells, a human acute monocytic leukemia cell line obtained from ATCC, were cultured in Roswell Park Memorial Institute (RPMI) medium (AL162S). This medium was enriched with 10% fetal bovine serum (FBS) and 0.05 mM of 2-mercaptoethanol, creating an optimal environment for cellular proliferation and metabolic activities. The monocytes were then differentiated into macrophages using phorbol 12-myristate 13-acetate (PMA), a potent stimulus that effectively induces cell differentiation. In parallel, other cell lines were also employed, including human epithelial kidney (HEK) cells, human colorectal adenocarcinoma cells (Caco-2), human alveolar basal epithelial cells (A549), Vero cells (derived from the kidney epithelial cells of African green monkey), and C6/36 cells (derived from *Aedes albopictus* mosquito larvae). These cells were obtained from ATCC and cultured in Dulbecco’s Modified Eagle Medium (DMEM) (Cat no: AL007A). This culture medium was supplemented with 10% FBS, 100 units/mL penicillin G sodium, and 100 µg/mL streptomycin sulfate to prevent bacterial contamination and to promote cell growth and proliferation. Each cell line was maintained under optimal conditions to ensure its viability and functionality for subsequent experimental procedures.

### Differentiation of macrophages

THP-1 monocytes were seeded in a 6-well plate supplemented with 100 ng/mL of phorbol 12-myristate 13-acetate (PMA). After a 24-hour incubation, the media was replaced by fresh RPMI media and incubated for 24–48 h for fully differentiated macrophages.

### Viruses and infection

The study utilized the CHIKV strain with the GenBank accession number JF950631.1 (66). Initial propagation of the virus was achieved in C6/36 cells, a mosquito cell line commonly used for arbovirus propagation. Firstly, C6/36 cells were seeded in a 6-well plate. Upon reaching an appropriate confluency, the C6/36 cells were infected with the CHIKV stock. The viral propagation period extended to 96 h (about four days) post-infection (hpi), after which the virus-containing medium was harvested. This harvested viral stock was subsequently used for infection experiments in human THP-1 cells, post-quantification via a plaque assay, a standard virological technique to determine viral titers. For the infection experiments, THP-1 cells were seeded into a 6-well plate and subjected to differentiation into macrophages via PMA activation. Forty-eight hours after PMA-mediated activation, the differentiated THP-1 macrophages were ready for infection. The CHIKV infection was conducted at a multiplicity of infection (MOI) of 1, ensuring that each cell received about one viral particle. The virus was diluted in serum-free media to facilitate efficient infection. At 2 hpi, the initial inoculum was removed, and the cells were washed to eliminate unbound virus particles. The medium was then replaced with RPMI media supplemented with 2% FBS, 1% penicillin/streptomycin, and 1% L-glutamine, providing a controlled environment for subsequent viral replication and cellular responses. At predefined time points post-infection, cells were harvested for different experiments.

### Plaque assay

Plaque assays were performed to detect the viable virus in the THP-1 cell supernatant using a 96-well format (67).

### Cloning of CHIKV 3′-UTR, host miRNAs, and miR-122b-5p targets

To comprehensively identify mature microRNAs (miRNAs), all mature miRNA sequences were aligned against the human reference genome using the Bowtie algorithm, a highly efficient short read aligner. To secure the precursor miRNA (pre-miRNA) sequences, 250 nucleotides upstream and downstream of each aligned mature miRNA were extracted. This extraction was accomplished through the application of custom Perl scripts, which enabled automated and accurate retrieval of the desired genomic sequences. Following the sequence mapping and extraction, each identified pre-miRNA was amplified from human RNA samples through polymerase chain reaction (PCR) using primers designed specifically to flank the extracted pre-miRNA sequences. The resultant PCR products, representing the pre-miRNA sequences, were then cloned into a pmR-mCherry vector, obtained from Clontech Laboratories, Inc. This vector serves as a carrier or “cloning vehicle,” allowing the pre-miRNA sequences to be stably maintained and propagated within a bacterial host. Parallelly, the 3′-untranslated region (3′-UTR) of the CHIKV was amplified from CHIKV RNA, using primers specifically designed to target the UTR region. The amplified CHIKV 3′-UTR was subsequently cloned into the pmirGLO vector (48). Additionally, potential cellular target sequences of hsa-miR-122b-5p were amplified from human RNA samples and cloned into the pmirGLO vector (Promega). The primers list is detailed in Table S3. This vector, which harbors both Firefly and Renilla luciferase genes, allows for dual-luciferase reporter assays, facilitating the quantitative evaluation of miRNA-mRNA interactions. The constructed vectors can then be transfected into cells to assess the regulatory impact of hsa-miR-122b-5p on its potential target sequences, providing valuable insights into the miRNA’s role in CHIKV infection using luciferase assay.

### Site-directed mutagenesis

The binding sites of hsa-miR122b-5p on CHIKV 3′-UTR were analyzed using RNA hybrid tool (68) (Version 2.2.1). For introducing changes at the binding site of miR-122b, we designed the internal primer of 3′-UTR consisting of modified nucleotides at desired positions and amplified it by two-step PCR. The amplified UTR containing mutated binding sites was cloned in pmirGLO vector for luciferase assay.

### Transfections

miR-122b-5p and miR-584 were individually transfected into HEK cells using the Jet PRIME transfection reagent, according to the manufacturer’s specifications. After the transfection, the cells were infected with CHIKV at a multiplicity of infection (MOI) of 1. Twenty-four hours post-infection, cells were harvested and subjected to RNA isolation, followed by quantitative real-time polymerase chain reaction (qRT-PCR) to quantitatively assess the viral genomic RNA.

For luciferase assay, HEK-293T cells were co-transfected separately with pmirGLO plasmids expressing the 3′-UTR of CHIKV and pmcherry vectors expressing the host miRNAs, using the Jet PRIME transfection reagent. After a 24-hour incubation period post-transfection, the cells were lysed and processed for the luciferase reporter assay, enabling an evaluation of the miRNA-mediated regulation of CHIKV 3′-UTR. Moreover, pmirGLO plasmids individually expressing various cellular targets of miR-122b-5p (namely, HDAC4, PAX8, KLHL17, DOCK7, NME3, GADD45B, DUSL3, and JOSD2) were co-transfected with pmcherry vector expressing miR-122b-5p into HEK-293T cells. At 24 h post-transfection (hpt), the cells were subjected to luciferase assays to ascertain the regulatory effects of miR-122b-5p on these potential target genes.

In another aspect of the study, miR-122b-5p mimic, antagomiR, cel-231 (negative control), scrambled miRNA, HDAC4-specific siRNA, and mock siRNA were synthesized by Invitrogen and transfected into PMA-activated THP-1 cells using Jet PRIME transfection reagent, at a final concentration of 100 pmol per well (6-well plate format). For experiments involving miR-122b-5p mimic and antagomiR, as well as their respective controls (cel-231 and scrambled miRNA), cells were incubated for 24 h post-transfection (hpt), followed by CHIKV infection at a multiplicity of infection (MOI) of 1. Cells were then harvested at 24 h post-infection (hpi) for RNA isolation, Western blot analysis, or confocal microscopy to assess the impact of miR-122b-5p modulation on CHIKV infection. For HDAC4 knockdown experiments, cells were collected at 12 hpi to capture the peak silencing efficiency of HDAC4 siRNA, as the knockdown effect was found diminished beyond this time due to siRNA degradation. These time points were selected based on prior optimization to ensure maximum knockdown efficiency and to minimize off-target variability, enabling a more accurate evaluation of downstream effects.

### Luciferase assay

Twenty-four hours post-transfection, HEK-293T cells were thoroughly rinsed with ice-cold phosphate-buffered saline (PBS) to remove any residual media and non-adherent cells. Following this, the cells were subjected to a luciferase reporter assay to measure the activities of Renilla and Firefly luciferases. This assay was conducted employing the Dual-Glo luciferase reporter assay system (Promega) in accordance with the manufacturer’s instructions. The luminescence signals were captured and quantified using a GloMax 20/20 Luminometer (Promega). For normalization purposes and to account for potential variations in transfection efficiency, the luminescence of Renilla luciferase was normalized to that of Firefly luciferase for each condition. This dual-luciferase system provides a robust and reliable approach to assess the relative impact of miRNA binding to its target sequences. To ensure the specificity of miR-122b-5p binding to the CHIKV 3′-UTR and host cellular targets, specific negative control conditions were established. An empty pmR-mCherry vector, along with the CHIKV 3′-UTR cloned into the pmirGLO vector, was used as a negative control to evaluate the binding of miR-122b-5p to the CHIKV 3′-UTR. In the absence of miR-122b-5p, no alteration in the Renilla luciferase activity would be expected. Similarly, for evaluating the binding of miR-122b-5p to host cellular targets, miR-122b-5p cloned into the pmR-mCherry vector was co-transfected with an empty pmirGLO vector. This setup would serve as a negative control, providing a baseline Renilla luciferase activity in the absence of potential host target sequences. In essence, these control conditions aid in discerning the specific effects of miR-122b-5p binding to the CHIKV 3′-UTR and host cellular targets from any non-specific effects or background noise.

### Biotinylated pull-down assay

In this study, we implemented a biotinylated pull-down assay following the established protocol (69). Briefly, streptavidin magnetic beads were initially prepared by incubating them with a blocking solution containing bovine serum albumin (BSA) and yeast tRNA. This blocking step was carried out overnight at 4°C to prevent non-specific binding to the beads. After the incubation, the beads were thoroughly washed with a wash buffer to remove any unbound blocking agents. The mature forms of hsa-miR-122b and the control cel-miR-231 were synthesized with a biotin label at the 3′ end (Sigma-Aldrich). These biotinylated miRNAs were then transfected separately into THP-1 cells using Lipofectamine RNAiMAX transfection reagent, according to the manufacturer’s protocol. After 24 h of transfection, the cells were infected with CHIKV. At 24 hpi, the cells were harvested and lysed using a buffer containing RNase and protease inhibitors, which helped preserve RNA integrity and prevent protein degradation. To ensure that variations in RNA concentration did not affect the results, RNA abundance was normalized based on input RNA levels before proceeding with the pull-down. The lysates, which contained the biotinylated miRNAs, were then incubated with streptavidin beads for one hour at room temperature to facilitate binding of the biotinylated miRNAs to the beads. Following this incubation, the beads were washed with a wash buffer to eliminate unbound cellular components. The bound RNA, including the biotinylated miRNAs and their interacting RNAs, was extracted from the beads using the Trizol method. The RNA abundance was measured using Qubit and normalized across all samples before the pull-down to ensure equal input levels. This step minimizes discrepancies due to variable RNA input concentrations followed by quantitative real-time PCR (qRT-PCR) to measure the levels of specific mRNAs. The qRT-PCR data were analyzed using the 2-ΔCt method to determine the relative expression levels of the mRNAs. We cross-validated fold changes using input normalization alongside the 2-ΔCt method to address potential exaggerations caused by enrichment. This biotinylated pull-down assay allows us to identify and quantify the mRNA targets of hsa-miR-122b in the context of CHIKV infection.

### miRNAs and transcripts expression profiling using qRT-PCR

For miRNA expression profiling, total RNA was extracted from cells using the TRIzol method. cDNA synthesis and PCR amplification were performed using the TaqMan Advanced cDNA Synthesis Kit and TaqMan Advanced miRNA Assays (Thermo Fisher Scientific), according to the manufacturer’s instructions. U6 snRNA was used as the endogenous control for miRNA normalization.

For transcript expression profiling, total RNA was used as a template for qPCR using a one-step SYBR Green quantitative real-time PCR kit (Thermo Fisher Scientific), with gene-specific primers listed in Table S3. GAPDH was used as the internal control. Fold changes were calculated using the 2^−ΔΔCt^ method.

For CHIKV RNA quantification, total RNA was extracted, and viral genomic RNA levels were quantified by qRT-PCR using CHIKV-specific primers. Viral RNA expression was reported as 100 × 2^−ΔCt^ method.

### Luminescence-based, high-throughput screening

For high-throughput screening, briefly, a library of 2042 miRIDIAN human miRNA mimics (miRbase Version19.0, Dharmacon) was replicated from stock plates to 384-well plates (CulturePlate-384, PerkinElmer). On the day of the screening, the library was reverse-transfected into HeLa cells (1,500 cells/well) using 0.1 µL/well of RNAiMAX (Thermo Fisher) at the final concentration of 50 nM. As negative controls, we used the miRIDIAN mimic negative controls 2 and 4, while as positive controls, we used two siRNAs specifically designed to target the CHIKV 3′-UTR. After 24 h post-miRNA transfection, one set of plates was transfected with pmiR-GLO-3′UTR and the other one with pmiR-GLO-empty as control. One hundred nanograms of plasmid DNA was transfected into each well using 0.3 µL of FugeneHD transfection reagent according to the manufacturer’s instructions. At 48 h post-plasmid DNA transfection, medium was completely aspirated, and 20 µL of Glo-lysis buffer (Promega) was added. Firefly and Renilla luciferase activities were measured using the Dual-Glo luciferase assay (Promega) system according to manufacturer instructions. The Firefly luciferase expression represents the assay output, where miRNA binding to the CHIKV 3′-UTR modulates Firefly luciferase activity. Renilla luciferase expression is included as an internal control to normalize for transfection efficiency.

The data were further analyzed using the R (Bioconductor) package “cellHTS2.” To identify the miRNAs having binding sites on CHIKV 3′-UTR, we took FLuc/RLuc ratio for each sample, i.e., miRNA mimics, positive control, and negative control, and compared the values among each other. The miRNAs with their ratios falling in the range of values of negative control were discarded. Secondly, we increased the stringency and discarded all the miRNAs in the overlapping region of negative control and positive control. Finally, we considered miRNAs with the values in the range of only positive control.

### RNA seq library preparation and sequencing

Total RNA of each sample (uninfected, 24 hpi, 36 hpi, 42 hpi, and antagomiR transfected) was extracted from cells using Trizol method. The quality and quantity checks of extracted RNA samples were done using Nanodrop 2000 Spectrophotometer (Thermo Fisher Scientific) and Bioanalyzer/Tape Station 4200 (Agilent Technologies). Library preparation of samples was done using Truseq Stranded mRNA Kit (Illumina) according to the manufacturer’s protocol, which generates mRNA-focused sequencing libraries from total RNA. Briefly, the protocol is discussed in our previous article (70).

### RNA seq analysis and identification of targets

Quality checks were performed using FastQC (Version 0.11.9) (http://www.bioinformatics.babraham.ac.uk/projects/fastqc/), and reads with scores > 20 were retained. Adapters were removed using Cutadapt (Version 3.4). We aligned the raw reads to the human reference genome assembly (GRCh38.p13) using STAR (Version 2.7.3a), and the GTF file (GRCh38.p13) was used for annotating transcripts/genes. We used the featureCounts tool (Version 2.0.1) to count reads per gene. The read counts were used to perform differential gene expression analysis using the Bioconductor package edgeR (Version 3.36.0). Briefly, the control and infected samples were compared at each time point to obtain log2 fold change (log2FC) and corresponding *P*-values for each gene. A similar analysis was also performed to compare antagomiR-transfected samples against the control cells, and significantly regulated genes were identified with a corrected *P*-value (FDR) of <0.05 and a log2 fold change (log2FC) threshold of ±1.5, where a positive change denotes upregulation and a negative change indicates downregulation. Commonly regulated genes across all libraries were taken, and miRNA targets were identified.

### Western blot

Cells were lysed in RIPA buffer and performed protein analyses using standard Western blotting protocol. Briefly, proteins on SDS-PAGE gels were transferred to nitrocellulose membranes followed by 1-h incubation in 5% BSA in PBS at room temperature. The following antibodies were used: HDAC4 (Santa Cruz, sc-46672 HRP, 1:8000 dilution) and β-actin sc-47778 HRP (1:6000) as primary antibodies. Membranes were incubated overnight at 4°C on a shaker. Post-washing, blots were incubated in anti-mouse secondary antibodies coupled with horseradish peroxidase (1:4,000) for 1 h at room temperature. Furthermore, blots were washed, and signals were detected with Chemidoc MP imaging system (Bio-Rad) using Super Signal West Pico PLUS Substrate (34577, Thermo Fisher Scientific). All washing processes were done using PBST.

### Confocal imaging

THP-1 cells were seeded on glass coverslips in 6-well plates with 100 ng/mL PMA for macrophage activations. Post-activation, we performed several transfections and CHIKV infections in different conditions (mimics-miR-122b-5p/cel-miR-231 transfection followed by infection, antagomiR-miR-122b-5p/scrambled miRNA transfections followed by infection and different time points of CHIKV infection). Post-treatment, cells were fixed with 4% paraformaldehyde in PBS for 15 min and then permeabilized for 30 min in 100% chilled methanol at 4°C. After washing with PBS, cells were incubated with blocking buffer (1%BSA and 0.3% Triton X-100 in PBS) for 45 min, followed by overnight incubation at 4°C with appropriate primary antibodies diluted in 1% BSA and 0.5% Triton X-100 in PBS. The primary antibodies were used: HDAC4 rabbit monoclonal antibody (Novus Biologicals, NBP2-16793, 1:100 dilution), IRF3 rabbit monoclonal antibody (Cell Signaling Technology, 4302S, 1:100 dilution), IRF3 (phospho-S386) rabbit monoclonal antibody (Abcam, ab76493, 1:100), and CHIKV-E1 mice antibody (in-house generated, 1:100). Further, AlexaFluor 591/488-conjugated secondary antibodies (Invitrogen) were added for 1 h. Then, the coverslips were washed three times with PBS and stained with DAPI (Invitrogen). Images of the samples were taken using a Nikon confocal microscope.

### Fluorescence intensity quantification

To evaluate the subcellular distribution of specific protein, we performed fluorescence intensity quantification using raw confocal microscopy .lif files. Images were opened in Fiji/ImageJ with Bio-Formats importer. For each condition, 40–50 cells were manually selected based on DAPI staining to delineate nuclear and cytoplasmic regions of interest (ROIs). Separate ROIs were drawn around the nucleus (blue channel) and cytoplasm on the corresponding IRF3, p-IRF3, and CHIKV-E1 channels (grayscale). Mean fluorescence intensity values were measured independently for nuclear and cytoplasmic compartments for each marker. Nuclear-to-cytoplasmic (N/C) ratios were calculated to assess subcellular localization dynamics. CHIKV-E1 cytoplasmic intensities were analyzed separately in the green channel. All imaging conditions (exposure, gain, and laser settings) were kept constant across all groups to ensure consistency.

### Measurement of chemokines and interleukins

The assay was performed using a standard protocol mentioned in Luminex Cytokine 35-Plex Human Panel (Thermo Fisher Scientific, Waltham, MA). The panel provides accurate and sensitive quantification of human proteins, such as EGF, Eotaxin, FGF-basic, G-CSF, GM-CSF, HGF, IFN-alpha, IFNgamma, IL-1 beta, IL-1 alpha, IL-1RA, IL-2, IL-2R, IL-3, IL-4, IL-5, IL-6, IL-7, IL-8, IL-9, IL-10, IL-12, IL-13, IL-15, IL-17A, IL17F, IL-22, CXCL10, CCL2, CXCL9, CCL3, CCL4, CCL5, TNFalpha, and VEGF. The fluorescence intensity was analyzed using the Luminex 100/200 instrument. The readings were normalized by standards, and the concentration of cytokines was measured using standard curve by curve fitting software.

### Correlation network analysis

To explore the association between modulated cytokines and immune-related genes, including interferon-stimulated genes (ISGs), a protein-protein interaction (PPI) network was constructed using the STRING database (version 11.5). The regulated cytokines and ISGs were input into the STRING platform to identify known and predicted interactions based on text mining, experimental data, and database annotations. The parameters for the analysis included a medium confidence interaction score (≥0.4), and the resulting network was visualized using Cytoscape (version 3.9.1). The network nodes represent the proteins (cytokines and ISGs), while the edges indicate interactions, with the line thickness reflecting the strength of data support.

### Statistics

Statistical analysis was conducted using GraphPad Prism (version 9). Data are presented as mean ± standard deviation (SD) or standard error of the mean (SEM), as indicated. All experiments included at least three independent biological replicates, and each imaging-based quantification was derived from at least three independent confocal sessions. For comparisons among multiple groups, one-way or two-way ANOVA was employed, followed by Tukey’s or Dunnett’s multiple comparisons test, as appropriate. Where applicable, false discovery rate (FDR) correction using the Benjamini–Hochberg method was applied to control for type I errors arising from multiple hypothesis testing (e.g., in ISG expression, cytokine profiling). Adjusted *P*-values, confidence intervals, and effect sizes were reported accordingly. Normality was assessed using the Shapiro–Wilk test prior to applying parametric tests. Paired *t*-tests were used, where intra-sample comparisons were necessary (e.g., cytokine data across paired treatment conditions). For viral RNA quantification, one-way ANOVA with Dunnett’s post hoc test was used to compare miR-122b-5p and miR-584-5p groups against the cel-231 transfection control. For confocal fluorescence intensity data, nuclear vs. cytoplasmic distributions of HDAC4, IRF3, and p-IRF3 were analyzed using two-way ANOVA, with post hoc corrections where applicable. Each bar in the corresponding figures includes individual cell data points for transparency. Functional enrichment analyses were assessed using hypergeometric tests or Fisher’s exact tests, with Benjamini–Hochberg FDR adjustment to determine significance. Significance thresholds were annotated as: *P* < 0.05 (*), *P* < 0.01 (**), *P* < 0.001 (***), *P* < 0.0001 (****); “ns” = not significant.

## Data Availability

Raw files are available in the GEO/SRA database under accession number GSE18656.

## SUPPLEMENTAL MATERIAL

Data Set S1 jvi.02118-24-s0001.xlsx Raw data of high-throughput miRNA screening to evaluate direct binding of human miRNAs to CHIKV 3′ UTR.

Data Set S2 jvi.02118-24-s0002.xlsx Detailed data of transcriptome sequencing of different libraries such as 24 hpi, 36 hpi, 42 hpi, and AntagomiR-122b.

Supplemental material jvi.02118-24-s0003.docx Figures S1 and S2, Tables S1 to S3, and uncropped Western image.

ASM does not own the copyrights to Supplemental Material that may be linked to, or accessed through, an article. The authors have granted ASM a non-exclusive, world-wide license to publish the Supplemental Material files. Please contact the corresponding author directly for reuse.
