# Supplementary material for: Dual regulatory role of hsa-miR-122b-5p in chikungunya virus infection via interaction with CHIKV 3′-UTR and HDAC4 modulation
Source: J Virol. 2025 Sep 16;99(10):e02118-24. doi: 10.1128/jvi.02118-24 (PMC12548407; doi:10.1128/jvi.02118-24)
Supplement: Supplemental material — Figures S1 and S2, Tables S1 to S3, and uncropped Western image. [file jvi.02118-24-s0003.docx]

**Dual Regulatory Role of hsa-miR-122b-5p in Chikungunya Virus Infection via Interaction with CHIKV 3'-UTR and HDAC4 Modulation**

Priyanshu Srivastava^1, @^, Nimisha Mishra^1^_,_ Sunil Kumar Dubey^1, $^, Jatin Shrinet^1, #^, Sakshi Chaudhary^1^, Ankit Kumar^1^, Ramesh Kumar^1^, Surbhi Malhotra^2^, Miguel Mano^3, 6^, Luca Braga^3, 5^, Binuja Varma^2^, Mauro Giacca^3, 4^, Sujatha Sunil^1*^

^1^Vector-Borne Diseases Group, International Centre for Genetic Engineering and Biotechnology (ICGEB), New Delhi, India

^2^Tata Consultancy Services, New Delhi

^3^Molecular Medicine Laboratory, International Centre for Genetic Engineering and Biotechnology (ICGEB), Trieste, Italy

^4^School of Cardiovascular Medicine & Sciences, King’s College London, London, United Kingdom

^5^Currently working in Functional Cell Biology Group, International Centre for Genetic Engineering and Biotechnology (ICGEB), Trieste, Italy

^6^Currently working in Center for Neuroscience and Cell Biology, Coimbra, PT

^@^Currently working in Department of Hematopoietic Biology and Malignancy, MD Anderson Cancer Center, Houston, TX, USA

^$^Currently working in Columbia University Medical Center, Columbia University, New York, NY, USA

^#^Currently working in Department of Biological Science, Florida State University, Tallahassee, FL, USA

Running Head: role of miR-122b-5p during CHIKV infection

* Address correspondence to [sujatha@icgeb.res.in](mailto:sujatha@icgeb.res.in)

**Supplementary information**

**
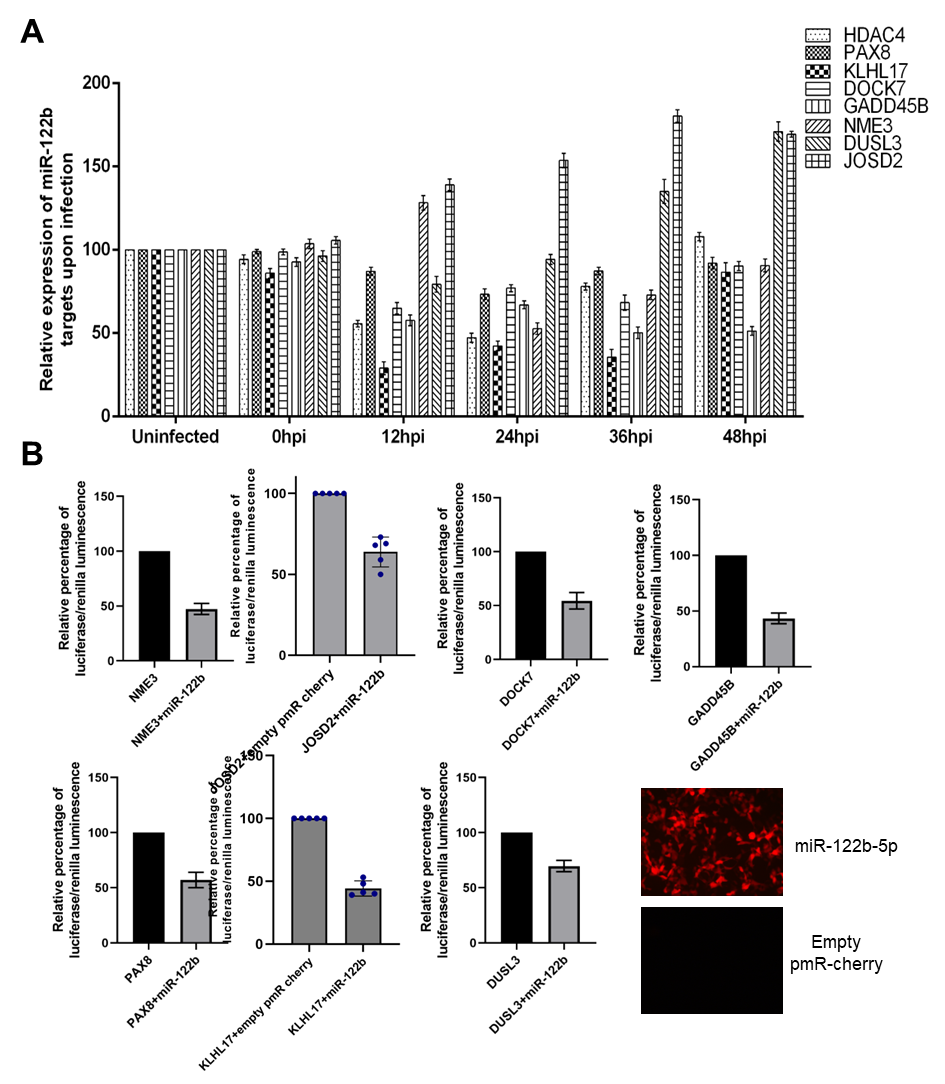
**

**Supplementary Fig.1: A.** Expression profiling of cellular targets of hsa-miR-122b-5p upon CHIKV infection in THP-1 macrophages. **B.** Luciferase assay showing the relative percentage of luciferase/renilla luminescence for miR-122b-5p effects on other cellular targets namely NME3, JOSD2, DOCK7, GADD45B, PAX8, KLHL17, and DUSL3. Results were plotted as mean firefly Luc activity (relative light units [RLUs]) standardized to control Renilla Luc activity ± SD. The experiments were repeated more than thrice, and each experiment included at least three treatments.


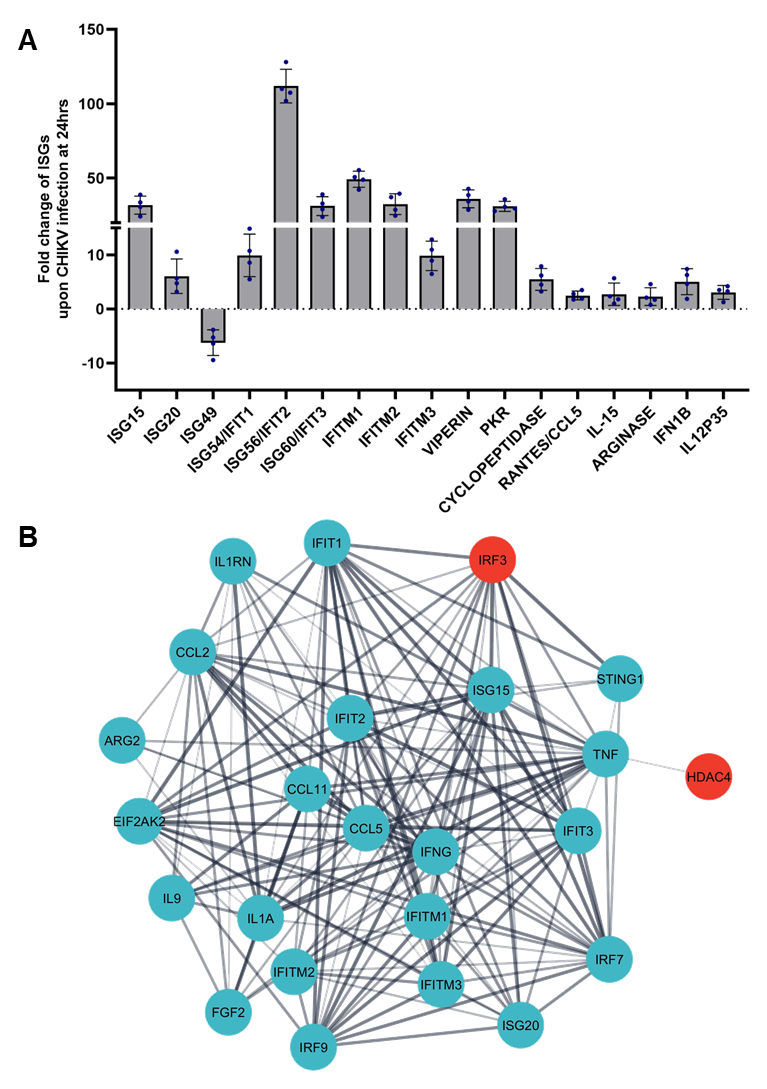


**Supplementary Fig.2: A.** Fold change in transcript expression level of Interferon Stimulating Factors (ISGs) upon CHIKV infection Data was analyzed using 2^-ΔΔCt^method and expressed as mean ± SD. Statistical significance was calculated by the One-way ANOVA method. **B.** Correlation network of modulated cytokines and immune-related genes, including ISGs, during CHIKV infection. The network highlights Interferon Regulatory Factor 3 (IRF3) as a central node connecting to several cytokines and ISGs, based on STRING database analysis. Other Interferon Regulatory Factors, such as IRF7 and IRF9, also appear in the network but were not further explored in this study. The edges indicate both functional and physical protein associations, with interactions sourced from Textmining, Experiments, and Databases. The line thickness represents the strength of the data support.

**Supplementary Table 1:** A total of 27 significantly regulated targets in AntagomiR-122b-5p library when compared with control and infection libraries. Red box shows the upregulated targets while blue box shows downregulated targets.

| **Gene stable ID** | **Transcript stable ID** | **Gene name** | **logFC** | **p_value** |
| --- | --- | --- | --- | --- |
| ENSG00000008988 | ENST00000009589 | RPS20 | 1.7818681 | 0.0028 |
| ENSG00000099860 | ENST00000215631 | GADD45B | 1.75332388 | 0.01085 |
| ENSG00000103024 | ENST00000219302 | NME3 | 1.58204537 | 5.00E-05 |
| ENSG00000119673 | ENST00000238651 | ACOT2 | 3.3175749 | 5.00E-05 |
| ENSG00000116641 | ENST00000251157 | DOCK7 | 2.77290223 | 0.0039 |
| ENSG00000125618 | ENST00000263334 | PAX8 | 2.01267763 | 1.00E-04 |
| ENSG00000154309 | ENST00000284476 | DISP1 | 1.56266525 | 5.00E-05 |
| ENSG00000168273 | ENST00000307106 | SMIM4 | 2.60221931 | 0.00055 |
| ENSG00000141994 | ENST00000309061 | DUS3L | 1.60844216 | 0.00385 |
| ENSG00000177337 | ENST00000317114 | DLGAP1-AS1 | 1.89836874 | 3.00E-04 |
| ENSG00000187961 | ENST00000338591 | KLHL17 | 1.81404529 | 0.00755 |
| ENSG00000068024 | ENST00000345617 | HDAC4 | 1.64264601 | 0.00805 |
| ENSG00000182841 | ENST00000357802 | RRP7BP | 1.54952742 | 0.0045 |
| ENSG00000225964 | ENST00000366140 | NRIR | 1.81227266 | 0.0188 |
| ENSG00000274998 | ENST00000391185 | SNORA17A | 3.66309788 | 5.00E-05 |
| ENSG00000215190 | ENST00000399751 | LINC00680 | 2.45578701 | 5.00E-05 |
| ENSG00000283710 | ENST00000408388 | MIR1204 | 2.33545339 | 5.00E-05 |
| ENSG00000224597 | ENST00000413405 | SVIL-AS1 | -1.581024 | 0.00425 |
| ENSG00000272419 | ENST00000418192 | LINC01145 | -1.6250723 | 0.00235 |
| ENSG00000224699 | ENST00000444238 | LAMTOR5-AS1 | -1.9415959 | 0.04485 |
| ENSG00000243679 | ENST00000450885 | | -2.5365001 | 0.0189 |
| ENSG00000225313 | ENST00000457957 | | -1.6528956 | 0.034 |
| ENSG00000240990 | ENST00000479766 | HOXA11-AS | -3.0167799 | 5.00E-05 |
| ENSG00000251562 | ENST00000508832 | MALAT1 | -2.9699512 | 0.0232 |
| ENSG00000259760 | ENST00000559569 | | -1.9324479 | 0.0204 |
|  |  |  | -2.3415158 | 0.01395 |
| ENSG00000161677 | ENST00000594350 | JOSD2 | -2.1413267 | 0.00127 |

**Supplementary Table 2:** Log2 fold change (Log2FC) of cytokine expression levels in different conditions: infection (Inf) vs. uninfected (Uninf) and AntagomiR+Inf vs. Inf. Statistical analysis was performed using a paired two-tailed t-test to assess the significance of the changes for each cytokine. Cytokines showing an "Opposite" trend are upregulated in the Inf vs. Uninf condition and significantly downregulated in the AntagomiR+Inf vs. Inf condition, highlighting their relevance in the context of AntagomiR treatment. Data are presented with Log2FC values and corresponding p-values, where p < 0.05 is considered significant.

| **Cytokine** | **Log2FC (Inf vs Uninf)** | **Log2FC (AntagomiR+Inf vs Inf)** | **p-value** | **Trend** |
| --- | --- | --- | --- | --- |
| IL-1 β | 1.2975 | 2.2136 | 0.0011 | Not Opposite |
| G-CSF (CSF-3) | 1.4491 | 2.4466 | 0.0015 | Not Opposite |
| IL-6 | 1.3162 | 5.4507 | 0.0021 | Not Opposite |
| IL-12/IL-23p40 | 0.0917 | 0.5035 | 0.0125 | Not Opposite |
| EGF | 0.9702 | 0.4556 | 0.0452 | Not Opposite |
| IL-1 α | 5.0614 | 1.5033 | 0.0013 | Not Opposite |
| IP-10 (CXCL10) | 2.2783 | 3.6906 | 0.0009 | Not Opposite |
| IL-2R | 0.9434 | 0.1769 | 0.0215 | Not Opposite |
| MIG (CXCL9) | 0.2839 | 0.8911 | 0.0143 | Not Opposite |
| IL-4 | 0.8758 | 0 | 0.0032 | Not Opposite |
| FGF-2 | 3.0110 | -0.3658 | 0.0001 | Opposite |
| IL-10 | 0.6914 | -0.1244 | 0.0047 | Opposite |
| RANTES (CCL5) | 3.1128 | -0.4725 | 0.0005 | Opposite |
| EOTAXIN (CCL11) | 0.7266 | -0.4716 | 0.0003 | Opposite |
| MIP-1 α (CCL3) | 2.1610 | -0.1447 | 0.0072 | Opposite |
| MIP-1 β (CCL4) | 2.4559 | -0.3862 | 0.0028 | Opposite |
| MCP-1 (CCL2) | 2.2741 | -1.0665 | 0.0002 | Opposite |
| VEGF-A | 4.2219 | -0.1408 | 0.0007 | Opposite |
| IFN-γ | 1.1160 | -0.4494 | 0.0004 | Opposite |
| IFN-α | 0.6895 | -0.1018 | 0.0069 | Opposite |
| IL-9 | 6.2788 | -0.5030 | 0.0008 | Opposite |
| IL-1RA | 3.7406 | -0.5722 | 0.0009 | Opposite |
| TNF α | 1.6003 | -1.6927 | 0.0006 | Opposite |
| IL-3 | 1.3020 | -0.4092 | 0.0054 | Opposite |

**Supplementary Table 3:** qPCR Primers of CHIKV gene, human interferon stimulating genes and miRNA’s host targets

| **Gene ID** | **PRIMER F** | **PRIMER R** |
| --- | --- | --- |
| CHIKV 3`UTR | ATGGAGCTCCTTGACAATTAAGTATGAAGG | ATGGTCGACGAAATATTAAAAACAAAATAA |
| IP_SDM_CHIKV 3`UTR | GATTGTCTTCTGCTTGAGTTAAACTCTTCATCCGTA |  |
| CHIKV E1 | TACCCATTTATGTGGGGC | GCCTTTGTACACCACGATT |
| HDAC4 | GCCAAAGATGACTTCCCTCTTA | CCGTCTTTCCTGCGTAACA |
| PAX8 | CACTCACCCTTCGCCATAAA | AAAGGCGGAGCTAGATAAAGAG |
| KLHL17 | AGGTTCTGGAACTGGTCTCTA | CGTCCACGTCGTGTTTCA |
| DOCK7 | GTGTCCTTGAGGCTGGATTT | GAGATGCAGGTGGAGTAAGTAAG |
| NME3 | CTCTGCCTCTTGGCCATT | CACGATGTTGATGTCGTTGTC |
| GADD45B | CACGCTCATCCAGTCCTTC | GTTCGTGACCAGGAGACAAT |
| DUS3L | GGATGTGACATGTGGAGAGATG | AGCTGGACGCCAAAGATG |
| JOSD2 | CGATGAGATCTGCAAGAGGTT | AGCGGCCATGATCACATT |
| ISG56 | CTTCAGGATGAAGGACAGGAAG | ACTTGGCTGCATATCGAAAGA |
| ISG54 | AAGGGTGGACACGGTTAAAG | ACTTGGCTGCATATCGAAAGA |
| ISG60 | CCATTGAGCTGAGTCCTGATAA | GCTTCTTCAACAAACTGCTCTC |
| RANTES | CTGCTGCTTTGCCTACATTG | ACACACTTGGCGGTTCTT |
| IL-15 | GCAATGAAGTGCTTTCTCTTGG | GTTGTTTGCTAGGATGATCAGATTT |
| ARGINASE II | CATGGACAGCCAGTTTCATTTC | CCACGTCTCTCAGACCAATATAC |
| IFN 1B | GCCGCATTGACCATCTATGA | GCCAGGAGGTTCTCAACAATAG |
| IL12p35 | GCCCTGTGCCTTAGTAGTATTT | GATCCATCAGAAGCTTTGCATTC |
| ISG15 | CTCTGAGCATCCTGGTGAGGAA | AAGGTCAGCCAGAACAGGTCGT |
| ISG20 | ACACGTCCACTGACAGGCTGTT | ATCTTCCACCGAGCTGTGTCCA |
| ISG49 | GCCTTGCTGAAGTGTGGAGGAA | ATCCAGGCGATAGGCAGAGATC |
| IFITM1 | GGCTTCATAGCATTCGCCTACTC | AGATGTTCAGGCACTTGGCGGT |
| IFITM2 | GGCTTCATAGCATTCGCGTACTC | AGATGTTCAGGCACTTGGCGGT |
| IFITM3 | CTGGGCTTCATAGCATTCGCCT | AGATGTTCAGGCACTTGGCGGT |
| VIPERIN | CCAGTGCAACTACAAATGCGGC | CGGTCTTGAAGAAATGGCTCTCC |
| PKR | GAAGTGGACCTCTACGCTTTGG | TGATGCCATCCCGTAGGTCTGT |

**Uncropped Western Image:** Protein expression profiling of HDAC4 upon CHIKV infection in THP-1 macrophages. we checked expression level of actin in same blot.

**Supplementary Data 1**: Raw data of high throughput miRNA screening to evaluate direct binding of human miRNAs to CHIKV 3`UTR.

**Supplementary Data 2:** Detailed data of transcriptome sequencing of different libraries such as 24hpi, 36hpi, 42hpi and AntagomiR-122b.
